# Effect of density functional approximations on the calculated Jahn–Teller distortion in bis(terpyridine)manganese(III) and related compounds

**DOI:** 10.1007/s00894-023-05812-0

**Published:** 2024-01-02

**Authors:** Jeanet Conradie

**Affiliations:** 1 Department of Chemistry, University of the Free State, P.O. Box 339, Bloemfontein, 9300 South Africa; 2 UiT - The Arctic University of Norway, N-9037 Tromsø, Norway

**Keywords:** Manganese(III), Jahn–Teller, DFT, Terpyridine

## Abstract

**Context:**

Bis(terpyridine)manganese(III) exhibits Jahn–Teller distortion due to the inequivalent occupation of the degenerate *e*_*g*_ orbitals of this high-spin d^4^ pseudo octahedral complex. Due to the spatially constrained nature of the terpyridine ligand, the central Mn-N bonds will always be shorter than the Mn-N terminal bonds, making it more difficult to distinguish between compression and elongation Jahn–Teller structures for bis(terpyridine)manganese(III). Density functional theory (DFT) calculations were utilized as a tool to evaluate the type of Jahn–Teller distortion in the high-spin d^4^ bis(terpyridine)manganese(III). The nature of the Jahn–Teller distortion calculated does depend upon the choice of density functional approximation (DFA) with the B3LYP, M06, and OLYP-D3 DFAs giving compression and the PW6B95D3, MN15, and MN15-D3 DFAs giving elongation in gas-phase calculations. All solvent-phase calculations yield an elongated structure for the bis(terpyridine)manganese(III) compound, which is yet to be structurally characterized experimentally. However, both gas and solvent OLYP-D3 calculations result in a compressed structure for the only experimentally isolated and characterized bis(terpyridine)manganese(III) complex, specifically the complex with terpyridine = 4′-(4-methylphenyl)-2,2′:6′,2′′-terpyridine. This alignment with the experimentally observed compression Jahn–Teller structure enhances the credibility of OLYP-D3 calculations in reproducing the observed geometries. The compressed Jahn–Teller geometries were near *D*_2d_ symmetry with the *z*-axis for compression defined along the Mn-N central bonds. Elongation Jahn–Teller distortion is not possible along the Mn-N central bonds, due to their spatially constrained nature. Thus, elongation occur along one pair of opposite Mn-N terminal bonds that are longer than the other pair of opposite terminal bonds, with shorter central bonds. The highest symmetry of the elongation Jahn–Teller distortion geometry of bis(terpyridine)manganese(III) is *C*_2v_. Criteria to distinguish between a compression and elongation Jahn–Teller geometry for bis(terpyridine)manganese(III) are identified. The nature of the singly occupied *e*_g_ molecular orbital, exhibiting anti-bonding interaction with the nitrogen-p MOs involved, dictates the type of Jahn–Teller distortion that occurs. The low-energy occupied bonding *t*_2g_ molecular orbitals establish bonds with and undergo mixing with the ligand molecular orbitals. The OLYP-D3 functional is recommended for calculating bis(terpyridine)manganese(III) and related compounds due to its consistent generation of metal–ligand bonds slightly longer than observed in experiments, in line with the required behavior. Additionally, OLYP-D3 offers a realistic electronic structure for Jahn–Teller distorted bis(terpyridine)manganese(III), correctly identifying alpha *e*_*g*_ molecular orbitals as the highest occupied molecular orbital and lowest unoccupied molecular orbital in agreement with experimental electrochemical studies. Furthermore, OLYP-D3 accurately reproduces the experimental compression geometry for the only structurally known bis(terpyridine)manganese(III) compound, instilling confidence in its reliability for such calculations.

**Methods:**

DFT geometry optimization and frequency calculations were done on the two different modes of Jahn–Teller distortion of bis(terpyridine)manganese(III), using the OLYP, B3LYP, M06, PW6B95D3, and MN15 functionals, with and without the Grimme’s D3 dispersion correction, and the 6-311G(d,p) or def2TZVPP basis set, as implemented in Gaussian 16. All optimizations were in the gas phase and also in the solvent phase with CH_3_CN as implicit solvent using IEFPCM.

**Graphical Abstract:**

DFT calculations were utilized to determine the Jahn–Teller effect on the geometry of high-spin d^4^ bis(terpyridine)manganese(III) complex containing two structurally constrained tridentate ligands.

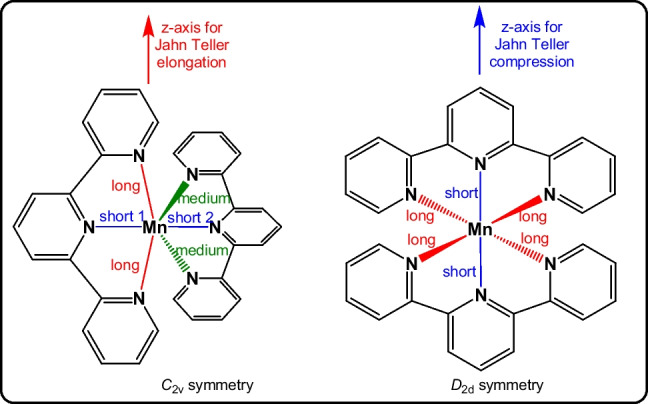

**Supplementary Information:**

The online version contains supplementary material available at 10.1007/s00894-023-05812-0.

## Introduction

**Scheme 1:**
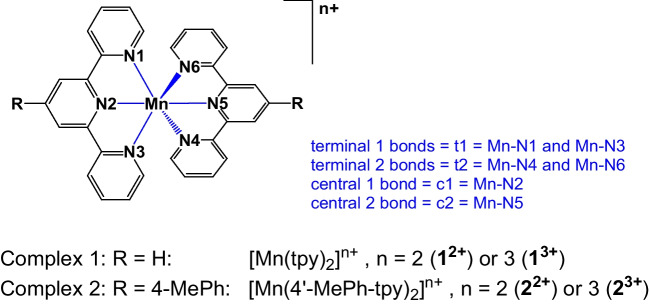
Structure, definition of bonds, and numbering of bis(terpyridine)manganese

Jahn–Teller distortions of octahedral transition metal complexes is an area of current interest as most such open-shell complexes show significant Jahn–Teller distortions which, in turn, are associated with multiple symmetry-equivalent potential energy surface minima [1]. Some complexes even show multiple different Jahn–Teller distortions, notably in the case of different ligands. This article concerns high-spin d^5^ manganese(II) and d^4^ manganese(III) complexes with two identical tridentate terpyridine ligands, see Scheme 1. Due to the reduced bite angles (ca 70–80°) of the tridentate 2,2′:6′,2″-terpyridine (tpy) ligands, these complexes have a distorted pseudo octahedral MnN_6_ coordination sphere.

**Fig. 1:**
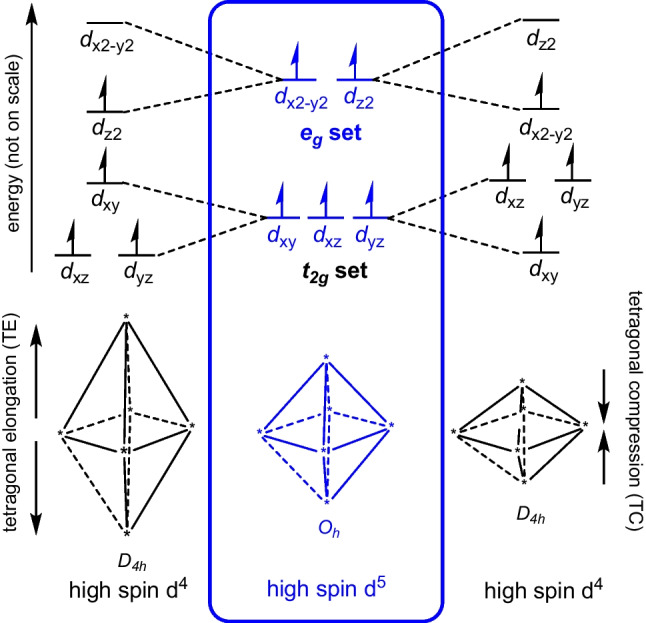
“Illustration of the effect of Jahn–Teller distortion on the geometry and orbital energies of high-spin d^4^ complexes, compared to high-spin d.^5^ octahedral (O_h_) complexes. *Z*-axis is defined in the vertical direction.” Reproduced from [4] (open access)

Jahn–Teller distortions in high-spin d^4^ octahedral complexes either result in compression or in elongation, due to the presence of a single electron in the *e*_*g*_ set of the d orbitals of high-spin d^4^ octahedral complexes [2–4], see Fig. 1. From an experimental point of view, which occurs depends upon the precise ligand [5]. Theory should, of course, not only reproduce the experimental result, but provide an explanation of why this result is observed. With the notable exception of a few CASPT2 or similar level ab initio calculations, almost all of computational studies take advantage of the highly efficient methodology of density functional theory (DFT) which implies a choice of density functional approximation (DFA). However, surprisingly little has been done to address the question of the dependence of the calculated distortion on the choice of DFA. The results in this manuscript show that the calculated Jahn–Teller distortion can indeed be different for different DFAs and goes on to show that this is intimately related to the order of the frontier molecular orbitals (MOs) produced by the DFA.

Bis(terpyridine)manganese coordination complexes contain a central manganese ion coordinated with two tpy ligands to form a stable structure in the case of manganese(II), complex 1^2+^ in Scheme 1. Bis(terpyridine)manganese(III), complex 1^3+^, in Scheme 1, that forms upon the oxidation of 1^2+^, however, is generally unstable, and with trace amounts of water loses a terpyridine to form a mixed-valent *di*-μ-oxo bridged binuclear complex [Mn_2_^III,IV^(O)_2_(terpyridine)_2_(H_2_O)_2_]^3+^ [6]. Numerous bis(terpyridine)manganese(II) complexes are known and isolated and characterized by solid-state crystallography [7] and other means [8–11], where terpyridine = the unsubstituted as well as substituted terpyridine ligands. However, only one bis(terpyridine)manganese(III) complex with terpyridine = 4′-(4-methylphenyl)-2,2′:6′,2′′-terpyridine (complex 2^3+^), is experimentally isolated and characterised by solid-state crystallography, as exhibiting a Jahn–Teller compressed pseudo octahedral geometry [12]. The novel complex 2^3+^ was obtained as a salt of ${\left({{\text{BF}}}_{4}\right)}^{-}$, ${\left({{\text{PF}}}_{6}\right)}^{-}$, and ${\left({{\text{ClO}}}_{4}\right)}^{-}$ after electrochemical oxidation of its Mn(II) complex, 2^2+^, under rigorous anhydrous conditions in CH_3_CN. Complex 2^3+^ is a rare example of a high-spin mononuclear Mn(III) complex stabilised solely by neutral nitrogen ligands [12]. Both DFT and CASSCF ab initio calculations predicted a positive sign of the zero-field splitting parameter *D* for 2^3+^ [12], in agreement with a compressed octahedral geometry [5, 13]. Attempts by the authors to isolate complex 1^3+^, after oxidation and bulk electrolysis of 1^2+^, failed. During the bulk electrolysis of 1^2+^, more than 2 e^−^ per molecule of 1^2+^ were consumed, resulting in unknown products.

Understanding and characterizing the Jahn–Teller effect in bis(terpyridine)manganese(III) complexes are important for the design and application of these complexes in various fields, including catalysis, magnetism, and molecular electronics. Due to the minimal experimental structural data available for bis(terpyridine)manganese(III) [12], a theoretical study on the Jahn–Teller distorted geometry of bis(terpyridine)manganese(III) is presented. Since DFT is the method of choice for computations by most workers in this field, it is important to know if and how the choice of DFA affects the nature of the calculated Jahn–Teller distortion. Complexes 1 and 2 (Scheme 1) are studied in two different oxidation states. The present work first validates different DFAs for their ability to describe the experimentally known geometry of 1^2+^ (i.e., compound 1 in the Mn(II) oxidation state). Results are found to be largely independent of DFA. Next, it is shown that the nature of the Jahn–Teller distortion of 1^3+^ does depend upon the choice of DFA with the B3LYP, M06, and OLYP-D3 DFAs giving compression and the PW6B95D3, MN15, and MN15-D3 DFAs giving elongation in gas-phase calculations. The reason for this DFA dependence is traced back to how the different functionals order the ligand field orbitals. Only elongation is observed in implicit solvent calculations for 1^3+^, consistent with what has been observed experimentally for most Mn(III) complexes. This validation is extended to 2^3+^ using those functionals which performed best for the 1^2+^ complex. Gratifyingly, the OLYP-D3 (gas-phase and solvent-phase) and PW6B95D3 (gas-phase) functionals gave compression Jahn–Teller geometries in good agreement with experimental solid-state X-ray structure [12].

## Theoretical methods

Chemcraft [14] was utilized to construct the input coordinates for density functional theory (DFT) calculations. All calculations were done using Gaussian 16 [15]. All molecules have undergone frequency analyses to guarantee the attainment of geometries with the lowest possible energy levels without any imaginary frequencies. The tight option for accurate optimizations and an ultrafine grid for integrals were applied. All optimizations were in the gas phase and also in the solvent phase with CH_3_CN as implicit solvent. The implicit solvent Integral Equation Formalism variant Polarizable Continuum Model was used (IEFPCM) [16, 17]. DFT calculations were done using a choice of DFAs:

(i) OLYP GGA (Generalized Gradient Approximation) functional [18, 19] with Grimme’s D3 dispersion correction [20] and the triple-ζ basis set 6-311G(d,p). The OLYP functional proved to correctly calculate the ${{d}_{{z}^{2}}}^{1}$ versus the ${{d}_{{x}^{2}-{y}^{2}}}^{1}$ ground state of the Jahn–Teller active d^4^ Mn(III) [21] and d^7^ Ni(III) [22].

(ii) B3LYP functional [19, 23] with basis set 6-311G(d,p). The B3LYP functional, in agreement with the OLYP [24], BP86 [25], and M06-L [25] functionals, proved to give the same Jahn–Teller ground state for high-spin d^4^ Mn(III) [25]. The B3LYP functional also proved to correctly calculate both the ${{d}_{{z}^{2}}}^{1}$ and ${{d}_{{x}^{2}-{y}^{2}}}^{1}$ ground states of the Jahn–Teller active high-spin d^4^ Cr(II) and low spin Cu(II) octahedral complexes [3].

(iii) M06 functional [26] with basis set 6-311G(d,p). The M06 [25] functional, in agreement with the OLYP [24], BP86 [25], and B3LYP [25] functionals, proved to give the same Jahn–Teller ground state for high-spin d^4^ Mn(III) [25].

(iv) PW6B95D3 [27] functional (6-parameter functional based on Perdew–Wang-91 exchange and Becke-95 correlation) of the Truhlar group, including the third order Grimme’s dispersion corrections [20] with the def2TZVPP basis set [28]. The PW6B95D3 hybrid meta exchange–correlation functional is chosen here, since it is developed for accurate thermochemistry, thermochemical kinetics, and non-bonded interactions [27].

(v) The MN15 Minnesota Functional of the Truhlar group [29, 30] with and without adding Grimme’s D3 dispersion correction [20] with the def2TZVPP basis set [28]. The MN15 functional is chosen here, since it has a broad accuracy in predicting a wide range of chemical properties with chemical accuracy. “The properties considered in the parameterization include bond energies, atomization energies, ionization potentials, electron affinities, proton affinities, reaction barrier heights, noncovalent interactions, hydrocarbon thermochemistry, isomerization energies, electronic excitation energies, absolute atomic energies, and molecular structures [29].”

Selected optimizations in the gas phase were done using ADF [31, 32] with the OLYP [18, 19] functional, adding Grimme’s D3 dispersion correction [20], using the scalar relativistic ZORA (zeroth order regular approximation to the Dirac equation) Hamiltonian [33–35] and the ZORA TZ2P all-electron relativistic basis set as implemented in ADF.

## Results and discussion 

Reported experimental results and a density functional theory (DFT) study on 1^2+^, utilizing different density functional approximations (DFAs), are firstly presented in subsection “Bis(terpyridine)manganese(II)”. The aim is to validate the chosen DFAs for their ability to describe the experimentally known geometry of 1^2+^. In subsection “Bis(terpyridine)manganese(III)”, the same DFAs are employed to determine the geometry and type of Jahn–Teller distortion in 1^3+^, as well as the character of the Mn-d MOs that predominantly contain the single electron in the e_g_ set of the d orbitals. This electron dictates the type of Jahn–Teller distortion that occurs (see Fig. 1). The DFT study is extended to the bis(terpyridine)manganese(II) complexes 2^2+^ and 2^3+^, which include a 4-methylphenyl substituent on the 4′ position of tpy (see Scheme 1), in subsection “Bis(4′-(4-methylphenyl)-2,2′:6′,2′′-terpyridine)manganese”. The selected DFAs are those that performed best for the 1^2+^ complex. In conclusion, subsection “Electronic structure of Mn(III)” evaluates the electronic structure of 1^3+^, considering the mixing of Mn-d and ligand MOs, and the ordering of the ligand field Mn-d MOs.

### Bis(terpyridine)manganese(II)

It is well reported in literature, on grounds of experimental reports [8–11] and theoretical calculations [36, 37], that the d^5^ bis(terpyridine)manganese(II), 1^2+^, is high spin with electronic configuration ${d}_{{\text{xy}}}^{1}{d}_{{\text{xz}}}^{1}{d}_{{\text{yz}}}^{1}{d}_{{x}^{2}-{y}^{2}}^{1}{d}_{{z}^{2}}^{1}$. The six nitrogen atoms of the terpyridine ligand have a pseudo octahedral arrangement around manganese, and due to the spatially constrained nature of the tridentate terpyridine ligand, the symmetry of high-spin bis(terpyridine)manganese(II) tetragonal system is not *D*_4h_ (ditetragonal dipyramidal) but *D*_2d_ (tetragonal scalenohedral). Most experimental structures of bis(terpyridine)manganese(II), 1^2+^, are near *D*_2d_ symmetry, see Table 1. Experimental terminal bonds vary between 2.28 and 2.22 Å with an average of 2.25(1) Å. Experimental central bonds vary between 2.22 and 2.18 Å with an average of 2.20(1) Å. Similar to the experimental structures, the DFT optimized geometries of bis(terpyridine)manganese(II), complex 1^2+^, are of *D*_2d_ or very near *D*_2d_ symmetry, see data in Table 2. The central bonds of the DFT optimized structures are between 0.04 and 0.06 Å (Δx2 in Table 2) shorter than the four terminal bonds (see definition of bonds in Scheme 1).

**Table 1:** Selected experimental Mn-N bond lengths (Å) for bis(terpyridine)manganese(II) (*S* = 5/2) as obtained from the Cambridge Structural Database (CSD [7, 38, 39]. Bonds are defined in Scheme 1

CSD refcode	t1	c1	t1	t2	c2	t2	*t*_ave_—*c*_ave_	*T* (K)
BINZAA	2.242	2.191	2.242	2.243	2.195	2.243	0.050	180
MEVSEL	2.27	2.191	2.238	2.235	2.181	2.25	0.062	RT
NIQRAI	2.247	2.196	2.234	2.239	2.198	2.262	0.048	RT
OKUHIN	2.246	2.205	2.253	2.255	2.215	2.281	0.049	100
OKUHIN	2.262	2.209	2.263	2.267	2.213	2.243	0.048	100
OTEWOA	2.267	2.186	2.244	2.272	2.192	2.244	0.068	RT
SIWFIN	2.216	2.19	2.26	2.254	2.194	2.264	0.056	153
XAHLIC	2.246	2.204	2.253	2.236	2.202	2.26	0.046	223
XENWAO	2.251	2.215	2.264	2.259	2.213	2.261	0.045	243
		Average	MAD^a^	Maximum	Minimum			
All terminal bonds:		2.252	0.011	2.281	2.216			
All central bonds:		2.199	0.009	2.215	2.181			

^a^*MAD* = mean absolute deviation (from the average value)

**Table 2:** Selected experimental and calculated bond lengths (Mn-N) for bis(terpyridine)manganese(II) (*S* = 5/2). Bonds are defined in Scheme 1. Calculated bond lengths are obtained with the indicated DFT functional. All lengths in Å

Method		Symmetry	Mn-N_t1_	Mn-N_t2_	Mn-N_c1_	Mn-N_c2_	Ave (Mn-N_c_)	Δx2^a^
Complex 1^2+^	Experimental ave ^b^	-	2.252	2.252	2.199	2.199	2.199	0.052
B3LYP	Gas	*D*_2d_	2.282	2.282	2.231	2.231	2.231	0.051
	Solvent	*D*_2d_	2.289	2.289	2.243	2.243	2.243	0.046
M06	Gas	*D*_2d_	2.232	2.232	2.203	2.203	2.203	0.029
	Solvent	*C*_1_	2.235	2.234	2.196	2.197	2.196	0.038
OLYP-D3	Gas	*D*_2d_	2.278	2.278	2.227	2.227	2.227	0.051
	Solvent	*C*_1_	2.288	2.288	2.227	2.227	2.227	0.061
	Gas^c^	*D*_2d_	2.253	2.253	2.237	2.237	2.237	0.016
PW6B95D3	Gas	*D*_2d_	2.257	2.257	2.216	2.216	2.216	0.041
	Solvent	*C*_1_	2.265	2.265	2.233	2.233	2.233	0.032
MN15	Gas	*D*_2d_	2.241	2.241	2.220	2.220	2.220	0.021
	Solvent	*C*_1_	2.240	2.239	2.212	2.213	2.213	0.027
MN15-D3	Gas	*D*_2d_	2.241	2.241	2.220	2.220	2.220	0.021
	Solvent	*C*_1_	2.240	2.239	2.212	2.213	2.213	0.027
Complex 2^2+^	Experimental ^d^		2.263	2.247	2.196	2.178	2.187	0.051
PW6B95D3	Gas	*C*_1_	2.260	2.260	2.198	2.198	2.198	0.062
OLYP-D3	Gas	*C*_1_	2.283	2.283	2.205	2.205	2.205	0.078
	Solvent	*C*_1_	2.292	2.292	2.216	2.216	2.216	0.076

^a^Δx2 = average{(Mn-N_t2_), (Mn-N_t1_)} – average{(Mn-N_c2_); (Mn-N_c1_)}

^b^Experimental average values for complex 1^2+^ from the data in Table 1

^c^TZ2P basis set

^d^Experimental values for complex 2^2+^ from reference [12]

When comparing gas and implicit solvent calculations with X-ray crystallography results for the solid, it should be noted that “chemical pressure” [40] in the crystal decreases metal–ligand bond lengths below calculated values in gas and implicit solvent models. Hence, the DFA should give longer calculated bond lengths than the experimental bond lengths. The DFT calculated metal–ligand terminal bonds for bis(terpyridine)manganese(II) as obtained by different DFAs (Table 2), all are within the range of the experimental bonds. Except for B3LYP that gave slightly longer metal–ligand central bonds, all DFT calculated central bonds are also within the range of the experimental bonds. Some of the M06 and MN15 calculated metal–ligand bonds are slightly shorter than the average of the experimental bonds, though still longer than the than some reported experimental metal–ligand bonds. Thus, all DFAs gave acceptable calculated metal–ligand bonds and a near *D*_2d_ geometry, in agreement with experiment. Results obtained here are thus largely independent of DFA. The OLYP-D3 and PW6B95D3 functionals, which consistently yielded slightly longer calculated metal–ligand bonds than the average experimental bonds in both gas- and solvent-phase calculations for 1^2+^, could be considered the best DFAs for calculations on the high-spin 1^2+^ complex.

### Bis(terpyridine)manganese(III)

In this section, we report results on bis(terpyridine)manganese(III), represented by complex 1^3+^ in Scheme 1, using the same DFAs as those employed for 1^2+^ in the previous section. We evaluate the geometry and type of Jahn–Teller distortion in 1^3+^, along with the character of the Mn-d MOs that predominantly contain the single electron in the *e*_*g*_ set of the d orbitals. This electron dictates the type of Jahn–Teller distortion that occurs (see Fig. 1).

**Fig. 2:**
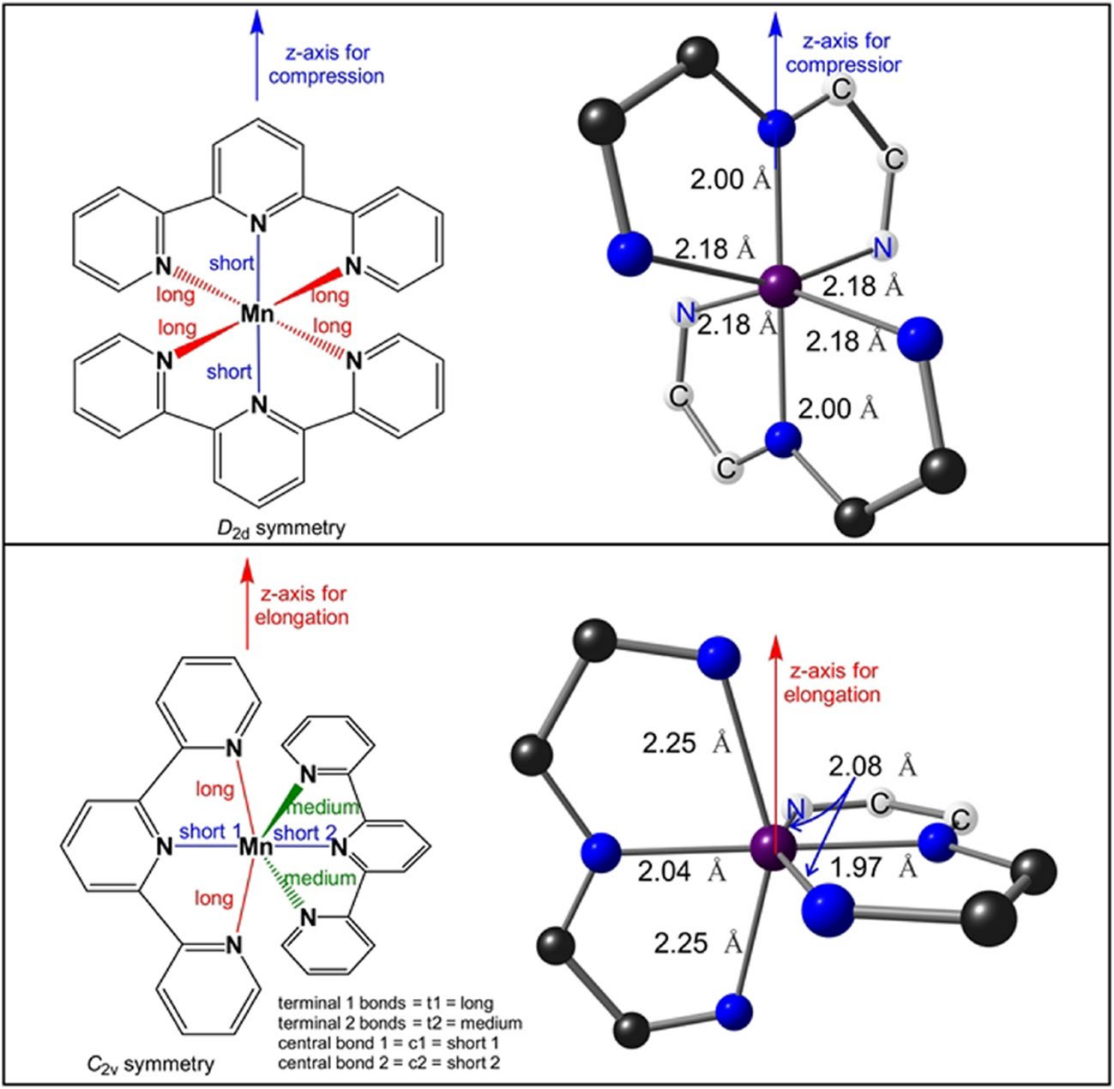
Jahn–Teller distortion for [Mn(tpy)_2_]^3+^, leading to elongation or compression along the *z*-axis as indicated. In the [Mn(tpy)_2_]^3+^ models on the right, the six-membered rings are excluded for clarity, with representative Mn-N bond lengths indicated. Mn, C, and N in purple, black, and blue, while the C and N to the backward direction are shown in white for perspective

Mn(III) complexes are generally high spin [2, 5, 12, 41, 42]. Similarly, the d^4^ bis(terpyridine)manganese(III), complex 1^3+^ in Scheme 1, is reported to be high spin on ground of both experimental reports [10, 12] and theoretical calculations [12, 36, 37], with an electronic configuration $\left({d}_{{\text{xy}}}^{1}{d}_{{\text{xz}}}^{1}{d}_{{\text{yz}}}^{1}\right)\left({e}_{g}^{1}\right)$. Like the high-spin d^4^ octahedral complexes shown in Fig. 1, the high-spin d^4^ complex 1^3+^ displays Jahn–Teller distortion, featuring a MnN_6_ coordination sphere that is distorted into a pseudo-octahedral shape, see Fig. 2. The only bis(terpyridine)manganese(III) type complex of which the structure is experimental determined by single-crystal X-ray crystallography, is complex 2^3+^ (Scheme 1), with a Jahn–Teller compressed geometry and electronic configuration ${d}_{{\text{xy}}}^{1}{d}_{{\text{xz}}}^{1}{d}_{{\text{yz}}}^{1}{d}_{{x}^{2}-{y}^{2}}^{1}{d}_{{z}^{2}}^{0}$ [12]. The single electron in the e_g_ set is thus in a molecular orbital of ${d}_{{x}^{2}-{y}^{2}}$ character (Fig. 1). However, theoretically both the ${d}_{{\text{xy}}}^{1}{d}_{{\text{xz}}}^{1}{d}_{{\text{yz}}}^{1}{d}_{{x}^{2}-{y}^{2}}^{1}{d}_{{z}^{2}}^{0}$ (*z*-in compressed geometry) and the ${d}_{{\text{xy}}}^{1}{d}_{{\text{xz}}}^{1}{d}_{{\text{yz}}}^{1}{d}_{{z}^{2}}^{1}{d}_{{x}^{2}-{y}^{2}}^{0}$ (*z*-out elongated geometry) electronic configurations are possible for complex 1^3+^, complex 2^3+^, and other bis(terpyridine)manganese(III) complexes containing substituted terpyridine ligands (Fig. 1).

#### Influence of DFA on geometry of bis(terpyridine)manganese(III)

Oxidation of bis(terpyridine)manganese(II) to bis(terpyridine)manganese(III), leads to a decrease in all the Mn-N bonds [12], see the Δ*d* values in Table 3. Mn(III) with a higher positive charge is more electron-deficient compared to Mn(II), leading to stronger electrostatic interactions between the metal and the ligands, resulting in shorter Mn-N bond lengths.

**Table 3:** Selected experimental and calculated bond lengths (Mn-N) for bis(terpyridine)manganese(III) (*S* = 2). Bonds are defined in Scheme 1. Calculated bond lengths are obtained with the indicated DFT functional. All lengths in Å. ave = average; Pg = point group

Method		JT	Pg	(Mn-N_t1_)_ave_	(Mn-N_t2_)_ave_	Mn-N_c1_	Mn-N_c2_	(Mn-N_c_)_ave_	Δx1 ^a^	Δx2 ^b^	Δd1^c^	Δd2^d^	Δd3^e^
Complex 1^3+^													
B3LYP	Gas	*z*-in	*C*_2v_	2.224	2.134	2.022	1.986	2.004	0.090	0.130	0.058	0.148	0.209
	Gas sp	*z*-out	*C*_1_	2.247	2.093	2.043	1.975	2.009	0.154	0.085	0.035	0.189	0.189
	Solvent	*z*-out	*C*_1_	2.247	2.093	2.043	1.975	2.009	0.154	0.085	0.042	0.195	0.201
M06	Gas	*z*-out	*D*_2d_	2.145	2.145	1.980	1.980	1.980	0.000	0.165	0.087	0.087	0.223
	Solvent	*z*-out	*C*_1_	2.206	2.065	2.018	1.959	1.989	0.141	0.076	0.028	0.169	0.178
OLYP-D3	Gas	*z*-out	*D*_2d_	2.179	2.179	2.001	2.001	2.001	0.000	0.177	0.099	0.099	0.226
	Gas sp	*z*-out	*C*_1_	2.242	2.097	2.041	1.976	2.009	0.145	0.089	0.036	0.181	0.186
	Solvent	*z*-out	*C*_1_	2.242	2.097	2.041	1.976	2.009	0.145	0.089	0.046	0.191	0.186
	Gas^f^	*z*-out	*C*_2v_	2.246	2.082	2.042	1.976	2.009	0.164	0.073	0.007	0.171	0.195
PW6B95D3	Gas	*z*-out	*C*_2v_	2.250	2.083	2.038	1.970	2.004	0.166	0.080	0.007	0.173	0.178
	Solvent	*z*-out	*C*_1_	2.262	2.061	2.054	1.963	2.009	0.201	0.052	0.003	0.204	0.179
MN15	Gas	*z*-out	*C*_2v_	2.207	2.053	2.019	1.954	1.986	0.154	0.067	0.034	0.188	0.201
	Solvent	*z*-out	*C*_1_	2.213	2.035	2.026	1.948	1.987	0.177	0.048	0.027	0.204	0.186
MN15-D3	Gas	*z*-out	*C*_2v_	2.208	2.053	2.019	1.954	1.987	0.154	0.067	0.033	0.188	0.201
	Solvent	*z*-out	*C*_1_	2.212	2.035	2.026	1.948	1.987	0.177	0.048	0.027	0.204	0.186
Complex 2^3+^	Experimental^g^	*z*-in		2.117	2.117	1.975	1.975	1.975	0.000	0.142	0.147	0.131	0.221
PW6B95D3	Gas	*z*-in	*C*_1_	2.171	2.129	1.964	1.948	1.956	0.042	0.173	0.089	0.131	0.234
OLYP-D3	Gas	*z*-in	*C*_1_	2.182	2.182	1.974	1.974	1.974	0.000	0.208	0.101	0.101	0.232
	Solvent	*z*-in	*C*_1_	2.180	2.180	1.986	1.986	1.986	0.000	0.194	0.112	0.112	0.230

^a^Δx1 = (Mn-N_t2_)_ave_—(Mn-N_t1_)_ave_

^b^Δx2 = (Mn-N_t2_)_ave_ – average{(Mn-N_c2_); (Mn-N_c1_)}

^c^Δd1 = (Mn-N_t1_)_Mn(II)_ – (Mn-N_t1_)_Mn(III)_

^d^Δd2 = (Mn-N_t2_)_Mn(II)_ – (Mn-N_t2_)_Mn(III)_

^e^Δd3 = (Mn-N_c,average_)_Mn(II)_ – (Mn-N_c,average_)_Mn(III)_

^f^TZ2P basis set

^g^Experimental values for complex 2^3+^ from reference [12]

When compression Jahn–Teller distortion occur, in going from bis(terpyridine)manganese(II) to bis(terpyridine)manganese(III), the decrease in the central Mn-N bonds, Δd3 in Table 3, are significantly more (> 0.20 Å) than the decrease in the terminal Mn-N bonds, Δd1 and Δd2 in Table 3, (< 0.15 Å). The compressed Jahn–Teller structure is of *D*_2d_ or very near *D*_2d_ symmetry with the *z*-axis for compression defined along the Mn-N central bonds, see Fig. 2. The compression Jahn–Teller distortion under *D*_2d_ symmetry is similar to the tetragonal compression Jahn–Teller distortion along the *z*-axis of an octahedron to *D*_4h_ symmetry (Fig. 1).

Elongation Jahn–Teller distortion is not possible along the Mn-N central bonds, due to their spatially constrained nature. Thus, elongation Jahn–Teller distortion for bis(terpyridine)manganese(III) needs to be along an opposite pair of terminal bonds, as illustrated in Fig. 2. The consequence is that one pair of opposite Mn-N terminal bonds (terminal bonds 1, t1) will be longer than the other pair of opposite terminal bonds (terminal bonds 2, t2). However, the t2 bonds will still be longer than both the central bonds. Thus, for elongation Jahn–Teller distortion, the t2 bonds will be of medium length compared to the t1 bonds and the central bonds, as illustrated in Fig. 2. Consequently, the Mn-N central bond (c1) between the long Mn-N terminal bonds 1 is slightly longer than the Mn-N central bond (c2) between the medium Mn-N terminal bonds 2. The highest symmetry of the elongation Jahn–Teller distortion geometry of bis(terpyridine)manganese(III) will thus be *C*_2v_. The elongation Jahn–Teller distortion under *C*_2v_ symmetry is similar to the orthorhombic elongation Jahn–Teller distortion of an octahedron [43].

Comparing the difference in bond length of Mn(III)-N_t2_ and Mn(III)-N_t1_ (Δx1 in Table 3) with the difference in bond length of Mn(III)-N_tl2_ and Mn(III)-N_c,average_ (Δx2 in Table 3), it is observed that for compression Jahn–Teller geometry, Δx1 is smaller than Δx2. In other words, the length of Mn(III)-N_t2_ is nearer to Mn(III)-N_t1_ than to Mn(III)-N_c,average_ for a compression Jahn–Teller geometry. Furthermore, the change in Mn-N_terminal2_ bond lengths upon reduction of Mn(III), Δd2 in Table 3, is smaller (< 0.15 Å) for compression than for elongation Jahn–Teller geometry (> 0.17 Å). Upon reduction of Mn(III) the Mn-N_central_ bond are more compressed for compression Jahn–Teller geometry (Δd3 in Table 3 > 0.2 Å) than for elongation Jahn–Teller geometry (Δd3 in Table 3 < 0.2 Å).

#### Influence of DFA on character of e_g_ MOs of bis(terpyridine)manganese(III)

**Fig. 3:**
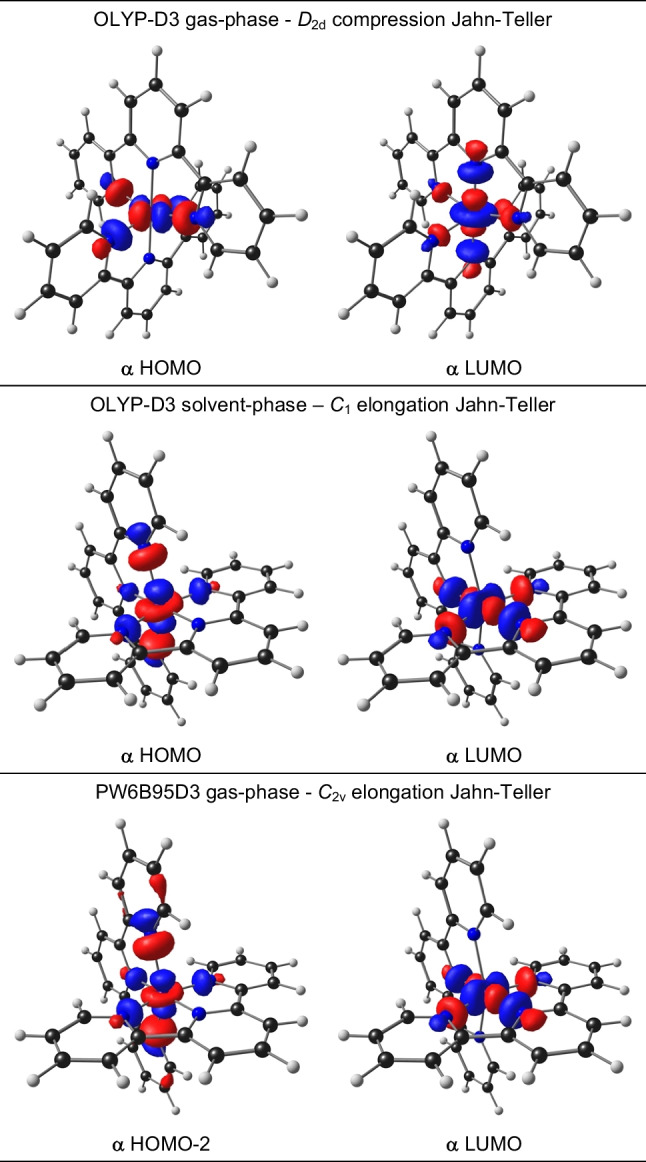
Selected α frontier orbitals for bis(terpyridine)manganese(III), obtained by the indicated DFT method. A contour of 0.06 Åe^−3^ was used for the MO plots. Color scheme used for atoms (online version): Mn (purple), N (blue), C (black), and H (white). The *z*-axis is in the vertical up direction as defined in Fig. 2

Gas-phase optimization, using the B3LYP, M06, or OLYP-D3 functionals, all gave a compressed Jahn–Teller structure for bis(terpyridine)manganese(III) with the *z*-axis for compression defined along the Mn-N central bonds, as shown in Fig. 2. The ${d}_{{x}^{2}-{y}^{2}}$ HOMO and ${d}_{{z}^{2}}$ LUMO (both α) of the OLYP-D3 results are shown in Fig. 3, confirming the electron occupation ${d}_{{\text{xy}}}^{1}{d}_{{\text{xz}}}^{1}{d}_{{\text{yz}}}^{1}{d}_{{x}^{2}-{y}^{2}}^{1}{d}_{{z}^{2}}^{0}$, expected for a compressed Jahn–Teller geometry (Fig. 1). On the contrary, gas-phase optimizations with the PW6B95D3, MN15, and MN15-D3 functionals gave an elongated Jahn–Teller structure for bis(terpyridine)manganese(III) with the *z*-axis for elongation defined along the longest Mn-N terminal bonds, as shown in Fig. 2. For these three functionals, the α MO of ${d}_{{z}^{2}}$ character is the α HOMO-2, while the LUMO (also α) is of ${d}_{{x}^{2}-{y}^{2}}$ character, see Fig. 3 for the gas-phase PW6B95D3 MOs, confirming the electron occupation ${d}_{{\text{xy}}}^{1}{d}_{{\text{xz}}}^{1}{d}_{{\text{yz}}}^{1}{d}_{{z}^{2}}^{1}{d}_{{x}^{2}-{y}^{2}}^{0}$, expected for an elongated Jahn–Teller geometry (Fig. 1).

Furthermore, when an implicit solvent (acetonitrile) was used for the optimization, all DFAs used gave an elongated Jahn–Teller structure for bis(terpyridine)manganese(III) with the *z*-axis for elongation defined along the longest Mn-N terminal bonds, as shown in Fig. 2. As example, the ${d}_{{z}^{2}}$ HOMO and ${d}_{{x}^{2}-{y}^{2}}$ LUMO (both α) of the OLYP-D3 solvent-phase results are shown in Fig. 3, confirming the electron occupation ${d}_{{\text{xy}}}^{1}{d}_{{\text{xz}}}^{1}{d}_{{\text{yz}}}^{1}{d}_{{z}^{2}}^{1}{d}_{{x}^{2}-{y}^{2}}^{0}$, expected for an elongated Jahn–Teller geometry (Fig. 1).

The % Mn and %N character of the ${d}_{{z}^{2}}$ and ${d}_{{x}^{2}-{y}^{2}}$ MOs of bis(terpyridine)manganese(III), obtained from the different DFAs, are provided in Table 4. The results show that irrespective if it is a Jahn–Teller elongation or compression geometry, the %Mn of the LUMOs are higher (50–55%) than the %Mn in the singly occupied *e*_*g*_ MO (28–32%, except for OLYP-D3 that is 47%).

**Table 4:** % Mn and %N character of the ${{\varvec{d}}}_{{{\varvec{z}}}^{2}}$ and ${{\varvec{d}}}_{{{\varvec{x}}}^{2}-{{\varvec{y}}}^{2}}$ MOs of bis(terpyridine)manganese(III) (*S* = 2), obtained with the indicated DFT functional. N are defined according to the bonds in Scheme 1 and Table 3. Pg = point group. For C_1_ point group, average values are given

Method		JT	Pg	α MO	Mn	N_t1_	N_t2_	N_c1_	N_c2_
Complex 1^3+^									
B3LYP	Gas	*z*-in	*C*_2v_	HOMO-1	31.71%	15.75%	8.30%	1.91%	-
				LUMO	55.39%	1.04%	5.39%	9.62%	11.39%
	Solvent	*z*-out	*C*_1_	HOMO-1	31.99%	14.62%	14.49%	-	-
				LUMO	55.36%	5.76%	5.60%	6.63%	9.52%
M06	Gas	*z*-in	*D*_2d_	HOMO	31.00%	12.25%	12.25%	-	-
				LUMO	53.51%	2.70%	2.70%	10.54%	10.54%
	Solvent	*z*-out	*C*_1_	HOMO	30.87%	14.72%	14.80%	-	-
				LUMO	53.55%	5.93%	5.92%	6.71%	9.35%
OLYP-D3	Gas	*z*-in	*D*_2d_	HOMO	47.30%	9.59%	9.59%	-	-
				LUMO	50.71%	2.95%	2.95%	11.07%	11.07%
	Solvent	*z*-out	*C*_1_	HOMO	46.65%	11.06%	11.19%	-	-
				LUMO	51.16%	6.07%	5.93%	7.31%	9.78%
PW6B95D3	Gas	*z*-out	*C*_2v_	HOMO-2	26.34%	18.36%	4.04%	3.93%	-
				LUMO	54.79%	-	6.87%	8.14%	11.44%
	Solvent	*z*-out	*C*_1_	HOMO-2	26.34%	18.36%	18.36%	-	-
				LUMO	54.79%	6.87%	6.87%	8.14%	11.44%
MN15	Gas	*z*-out	*C*_2v_	HOMO-2	28.42%	18.55%	3.67%	4.21%	-
				LUMO	54.33%	-	7.42%	8.14%	11.57%
	Solvent	*z*-out	*C*_1_	HOMO-2	27.67%	15.35%	18.55%	-	-
				LUMO	54.25%	6.06%	6.15%	5.52%	9.00%
MN15-d3	Gas	*z*-out	*C*_2v_	HOMO-2	28.41%	18.55%	3.67%	4.22%	-
				LUMO	54.33%	-	7.42%	8.14%	11.58%
	Solvent	*z*-out	*C*_1_	HOMO-2	27.66%	15.36%	15.35%	-	-
				LUMO	54.25%	6.06%	6.15%	5.52%	9.00%
Complex 2^3+^									
PW6B95D3	Gas	*z*-in	*C*_1_	HOMO-4	31.63%	13.28%	9.95%	1.17%	-
				LUMO	56.63%	1.45%	2.74%	10.21%	11.12%
OLYP-D3	Gas	*z*-in	*C*_1_	HOMO-2	50.93%	7.22%	7.23%	-	-
				LUMO	52.76%	-	-	8.61%	8.61%
	Solvent	*z*-in	*C*_1_	HOMO	48.67%	7.60%	7.60%	7.60%	7.60%
				LUMO	51.43%				

For the compression Jahn–Teller geometries, the singly occupied *e*_*g*_ MO is mainly located on Mn and on the four terminal nitrogen (8–16%) with less than 2% on the central nitrogens. The LUMOs of the compression Jahn–Teller geometries are located on Mn and 9–12% on the two central nitrogens, but less than 6% on the terminal nitrogens.

For the elongated Jahn–Teller geometries, the singly occupied *e*_*g*_ MO is mainly located on Mn and the two terminal nitrogens along the *z*-axis, N_t1_, with ca 18%N each and less than 4% on the nitrogens along the *x*- and *y*-axis (N_t2_ and the central nitrogens). The LUMOs of the elongated Jahn–Teller geometries are located on Mn and 6–12% on the nitrogens along the *x*- and *y*-axis (N_t2_ and the central nitrogens), but less than 1% on the two terminal nitrogens along the *z*-axis, N_t1_. The character of the singly occupied *e*_*g*_ MO and the LUMO is thus clearly either ${d}_{{z}^{2}}$ or ${d}_{{x}^{2}-{y}^{2}}$ and can be used to distinguish between an elongated or compression Jahn–Teller geometry.

As noted in Table 4, the order of the *e*_*g*_ Mn-d MO for 1^3+^ was the same, irrespective of it was a gas/solvent-phase calculation producing a *z*-in/z-out geometry. For example, B3LYP gas phase produce a *z*-in geometry and the solvent phase *z*-out geometry, but the *e*_*g*_ Mn-d MO was still the α HOMO-1. However, as will be pointed out in the next section, for OLYP-D3 calculation of 2^3+^, this was not true (Table 4). For the gas-phase *z*-in geometry of 2^3+^, the *e*_*g*_ Mn-d MO was the α HOMO-2, while for the solvent-phase *z*-in geometry it was the α HOMO.

#### Temperature effect on z-in/z-out population

**Fig. 4:**
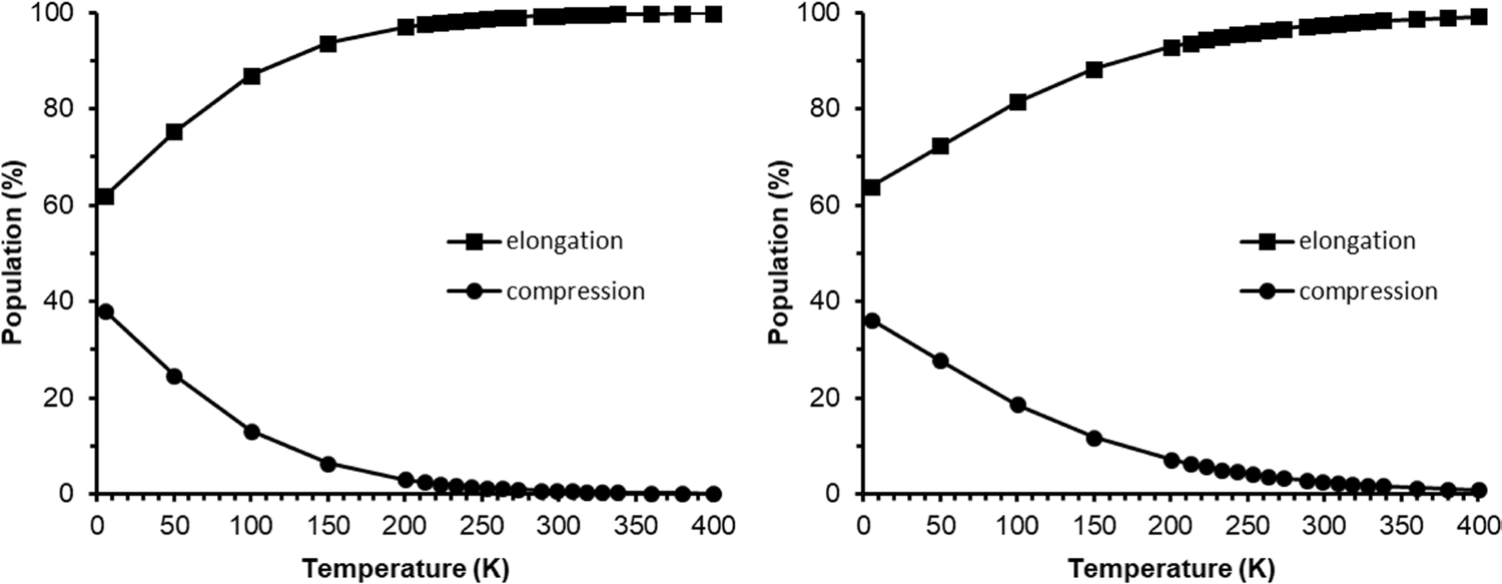
Gas-phase relative population as a function of temperature of the elongated and compressed Jahn–Teller geometries, calculated using the Boltzmann distribution. Left OLYP-D3 and right B3LYP results

It was not possible to optimize any elongated Jahn–Teller structure for bis(terpyridine)manganese(III) in the gas phase when using the functionals B3LYP, M06, and OLYP-D3. However, when using the solvent-phase optimized elongated Jahn–Teller structure of B3LYP and OLYP-D3 respectively and running a single point calculation on the solvent-phase optimized elongated Jahn–Teller structure in the gas phase, energies for an elongated Jahn–Teller structure in the gas phase could be obtained (the result did not have any imaginary frequencies), see Table 5. The electronic energies (*E*) of the single point calculation in the gas phase, on the solvent-phase optimized elongated Jahn–Teller structure, were slightly higher than the optimized gas-phase compression Jahn–Teller structure, explaining why no elongated Jahn–Teller structure could be obtained in the gas phase for these functionals. However, in contrary to the electronic energies, the elongated Jahn–Teller structures have slightly lower free energies (*G*) at 298 K. The free energies are temperature dependent, and by using the Boltzmann distribution, the relative population of the elongated and compressed Jahn–Teller structures as a function of temperature can be determined, see Fig. 4. It is clear that according to free energies, the elongated structure is favored, though the compression structure is also possible with a lower probability.

**Table 5:** Relative energies for bis(terpyridine)manganese(III) (complex 1^3+^, *S* = 2), obtained in the gas phase with the indicated functional

Method	JT	symm	Δ*E* (eV)	Δ*G* (eV) at 298 K
B3LYP	z-in	*C*_2v_	0.00	0.04
	z-out	*C*_1_	0.01	0.00
OLYP-D3	z-in	*D*_2d_	0.00	0.08
	z-out	*C*_1_	0.02	0.00

### Bis(4′-(4-methylphenyl)-2,2′:6′,2′′-terpyridine)manganese

In this section, DFT calculated results on the experimentally known complexes 2^2+^ (geometry) and 2^3+^ (geometry and *e*_*g*_ MOs) in Scheme 1, using two selected DFAs, are reported.

**Fig. 5:**
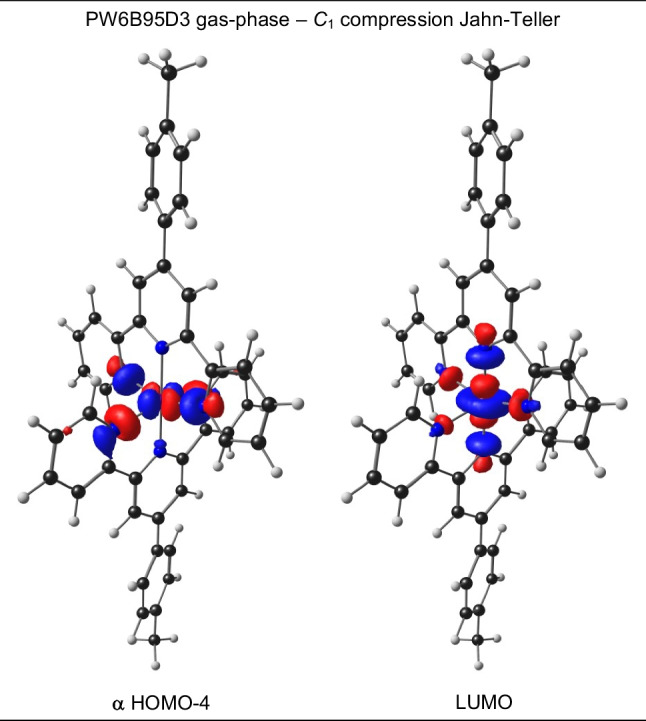
Selected α frontier orbitals for bis(4′-(4-methylphenyl)-2,2′:6′,2′′-terpyridine)manganese(III), obtained by the indicated DFT method. A contour of 0.06 Åe^−3^ was used for the MO plots. Color scheme used for atoms (online version): Mn (purple), N (blue), C (black), and H (white). The *z*-axis is in the vertical up direction as defined in Fig. 2

The OLYP-D3 functional, as well as the PW6B95D3 functional, that best reproduced the average experimental bonds for bis(terpyridine)manganese(II), complex 1^2+^, was chosen to optimize bis(4′-(4-methylphenyl)-2,2′:6′,2′′-terpyridine)manganese, complex 2, for which both the manganese(II) and compression Jahn–Teller manganese(III) structures are experimentally isolated and characterised by solid-state crystallography [12]. For OLYP-D3, both gas- and solvent-phase calculations were done, to determine the influence of the medium on the DFT results. Solvent calculation using PW6B95D3 were not considered for 2^3+^, since for 2^2+^ it gave shorter than experimental calculated metal–ligand bonds. The results provided in Table 2 and 3 show that the gas-phase DFAs reasonably reproduced the experimental structures of 2. The RMSD between the structure overlay of experimental and gas phase calculated are 0.63/0.61 and 0.53/0.51 Å (for PW6B95D3/OLYP-D3) for Mn(II) and Mn(III) respectively. Notably, OLYP-D3 produced calculated metal–ligand bonds slightly longer than those observed in the experiment, as required. Gratifying, in agreement with experiment, for both functionals, bis(4′-(4-methylphenyl)-2,2′:6′,2′′-terpyridine)manganese(III) optimized to a compression Jahn–Teller structure, see selected bond lengths in Table 3 and the MOs involved Fig. 5. It should be noted that complex 1^3+^, containing unsubstituted terpyridine ligands, optimized to an elongation Jahn–Teller structure when using the PW6B95D3 functional (Fig. 3). Thus, both PW6B95D3 (gas phase) and OLYP-D3 (gas and solvent phase) yielded similar accurate results, consistent with experimental findings. In Table 4, the %Mn and %N character of the ${d}_{{z}^{2}}$ and ${d}_{{x}^{2}-{y}^{2}}$ MOs of bis(4′-(4-methylphenyl)-2,2′:6′,2′′-terpyridine)manganese(III) are given. For both functionals the singly occupied *e*_*g*_ MO and LUMO are clearly of ${d}_{{x}^{2}-{y}^{2}}$ and ${d}_{{z}^{2}}$ character respectively. The ${d}_{{x}^{2}-{y}^{2}}$, SUMO has ca 32% (for PW6B95D3, ca 50% for OLYP-D3) are on Mn and 9–13% (for PW6B95D3, ca 7% for OLYP-D3) on each terminal nitrogen. The ${d}_{{z}^{2}}$ LUMO has ca 57% on Mn (for PW6B95D3, ca 52% for OLYP-D3) and ca 11% (for PW6B95D3, 7–8% for OLYP-D3) on the 2 central nitrogens.

For the OLYP-D3 gas-phase *z*-in geometry of 2^3+^, the *e*_*g*_ Mn-d MO was the α HOMO-2 (α HOMO-2 for gas-phase PW6B95D3), while for the OLYP-D3 solvent-phase *z*-in geometry, it was the α HOMO. In the next section, an explanation will be provided, based on experimental observations in a solvent environment, to support the assertion that the *e*_*g*_ Mn-d MO of Mn(III) should be the α HOMO.

### Electronic structure of Mn(III)

In this section, we explore the character, ordering, and mixing of the Mn-d and ligand MOs in bis(terpyridine)manganese(III). We compare experimental observations with DFT results to identify the most suitable DFA for accurately describing the nature of Jahn–Teller distortion in bis(terpyridine)manganese(III) and related compounds.

The only experimentally isolated Mn(III) complex containing two terpyridine ligands is 2^3+^, obtained upon the electrochemical oxidation of 2^2+^ [12]. The reduction of 2^3+^ showed Mn(III/II) reduction, as well as two ligand-based reduction peaks. The ligand-based reduction peaks occurred at the same potential as the ligand-based reduction peaks of 2^2+^ [12]. This is consistent with the HOMO of 2^3+^ being Mn-based, and the HOMO-1 and HOMO-2 of 2^3+^ being ligand-based [44], assuming no re-ordering of MOs occurs upon reduction [45]. Mn(II/III) oxidation and ligand-based reduction have been experimentally [8–12, 46] and theoretically [36, 37] reported for a series of different bis(terpyridine)manganese(II) complexes. The oxidation of 2^3+^ showed reversible manganese-based Mn(III/IV) oxidation [12]. This is consistent with the LUMO of 2^3+^ being Mn-based [44]. Thus, since the experimental oxidation and reduction of 2^3+^ are manganese-based, the HOMO and LUMO of 2^3+^ are expected to be mainly manganese-based [44], assuming no re-ordering of MOs occurs upon oxidation or reduction [45].

Based on this experimental evidence, the OLYP-D3 and M06 functionals, which provided mainly manganese-based HOMO and LUMO (Table 4), offer a more realistic electronic description of bis(terpyridine)manganese(III) than the other functionals reported in this work. In subsection “Bis(terpyridine)manganese(II)” for 1^2+^ (Table 2) and subsection “Bis(4′-(4-methylphenyl)-2,2′:6′,2′′-terpyridine)manganese” for 2^2+^ and 2^3+^ (Table 3), it was illustrated that the OLYP-D3 functional gave, as expected, slightly longer calculated metal–ligand bonds than experimental bonds, making the OLYP-D3 the recommended DFA to be used for describing both the geometry and electronic structure of bis(terpyridine)manganese(III) and related compounds.

**Fig. 6:**
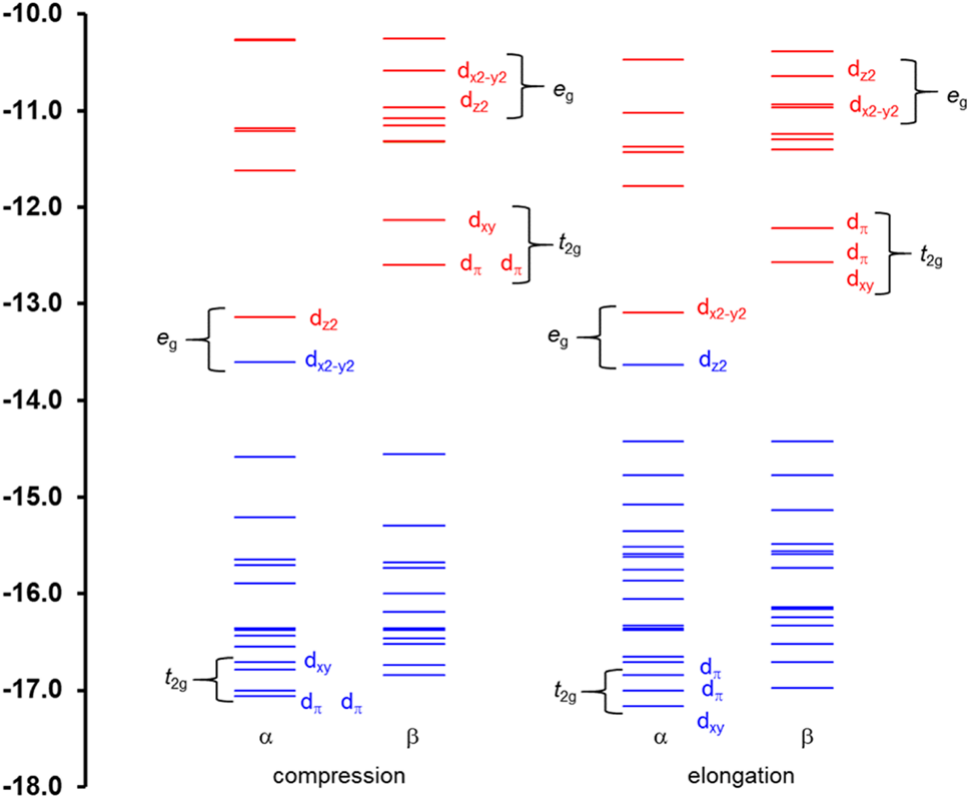
OLYP-D3 calculated MO energy level diagram for the top highest occupied and lowest unoccupied molecular orbitals of high-spin d^4^ bis(terpyridine)manganese(III). Blue and red lines show energies of occupied and unoccupied MOs respectively, *Y*-axis energy in eV

Therefore, to gain more insight into the electronic structure of bis(terpyridine)manganese(III), results obtained from the OLYP-D3 functional will be used, though the other functionals gave similar results. In Fig. 6, the energies of the top occupied and unoccupied MOs of the OLYP-D3 gas-phase-calculated elongation and compression geometries are graphically illustrated. An evaluation of the % Mn, N, and C contribution to the top highest occupied and lowest unoccupied molecular orbitals of the OLYP-D3 optimized geometries of 1^3+^ (both elongation and compression, see Tables S1 and S2) of bis(terpyridine)manganese(III) revealed that the HOMO (an *e*_*g*_ α MO) and LUMO (an *e*_*g*_ α MO), as well as and LUMO + 1 – LUMO + 3 (*t*_2g_ β-MOs) are mainly manganese based. These *e*_*g*_ and *t*_2g_ MOs are well separated from the other MOs. The α *e*_*g*_ MOs also exhibit an observable amount of nitrogen-p character, representing the anti-bonding interaction between the Mn-d and N-p MOs. The β *t*_2g_ MOs, on the other hand, have in addition to Mn-d, 25–40% ligand π character.

Below the HOMO are six pure ligand π based MOs (refer to Figure S1 for HOMO-1 – HOMO-6), followed by MOs that are ligand-based with a small contribution of Mn-*d*_π_ or Mn-*d*_xy_ (ca 10–20%) for some MOs (refer to Tables S1 and S2). In other words, mixing of ligand and Mn-d MOs occur. The mainly manganese-based occupied *t*_2g_ α MOs are much lower in energy (HOMO-30 and lower), and have in addition to the ca 50% Mn-d character, also ligand character.

Similarly, the mainly manganese based LUMO to LUMO + 3 (refer to Figure S1) are followed by mainly ligand π-based MOs. Some of these ligand-based MOs contain a small amount of Mn-d_π_ or Mn-*d*_xy_, as indicated in Table S1. The unoccupied mainly manganese-based *e*_*g*_ β-MOs, on the other hand, are of much higher energy (LUMO + 12 and higher, see Fig. 6), showing again mainly anti-bonding interaction between the Mn-d and N-p MOs.

In summary, the *e*_*g*_ MOs have mainly Mn-d and N-p character while the *t*_2g_ MOs mainly have Mn-d and ligand-π character. In addition, the ligand-based MOs also in some cases have a small amount of Mn-*d*_π_ or Mn-*d*_xy_, indicating mixing of the *t*_2g_ MOs with ligand MOs. This mixing of MOs is also clear when evaluated the density of states (DOS) and partial density of states (PDOS) of bis(terpyridine)manganese(III), see Figure S2.

**Fig. 7:**
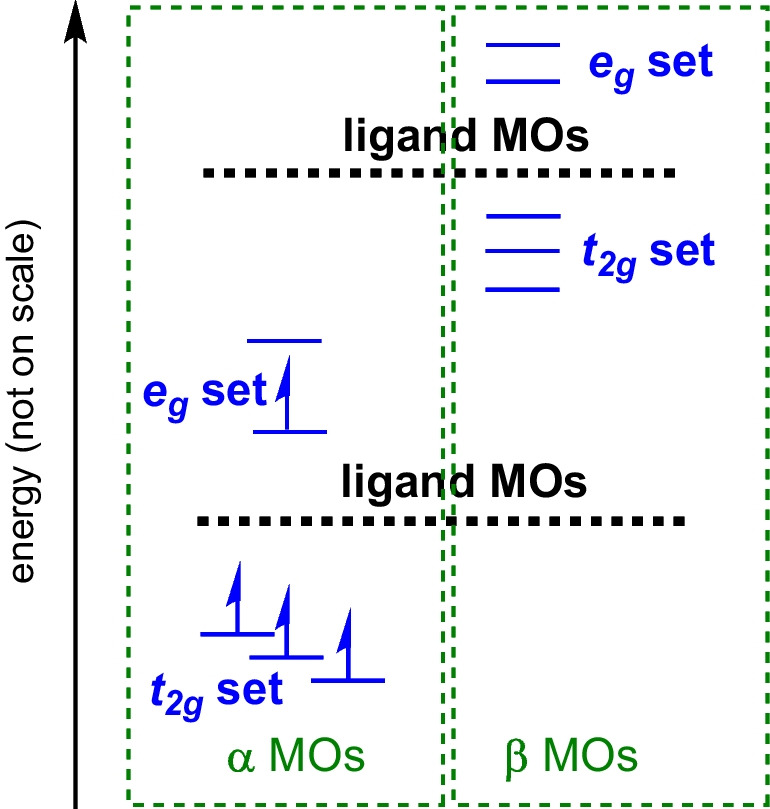
Generic MO energy level diagram for the top highest occupied and lowest unoccupied molecular orbitals of high-spin paramagnetic d^4^ bis(terpyridine)manganese(III)

The character of the top highest occupied and lowest unoccupied molecular orbitals of the open-shell high-spin d^4^ bis(terpyridine)manganese(III) reveals a generic MO energy level diagram depicted in Fig. 7, akin to what was found for closed-shell d^6^ bis(polypyridyl)ruthenium(II) complexes [47].

An interesting observation is that, for the Jahn–Teller distorted spatially constrained pseudo-octahedral geometry of d^4^ bis(terpyridine)manganese(III), the ordering of the *t*_2g_ MOs, specifically the *d*_π_ and the *d*_xy_ MOs (Fig. 6), is opposite to what is predicted for Jahn–Teller distortion of a real octahedral geometry (Fig. 1). Jahn–Teller distorted elongation and compression d^4^ bis(terpyridine)manganese(III) is of near *C*_2v_ and *D*_2d_ symmetry, while Jahn–Teller distorted elongation and compression of a real octahedral geometry is of *D*_4h_ symmetry. In bis(terpyridine)manganese(III), the character of the singly occupied *e*_*g*_ molecular orbital, which engages in an anti-bonding interaction with the nitrogen-p molecular orbitals, determines the specific manifestation of Jahn–Teller distortion. Concurrently, the low energy occupied bonding *t*_2g_ molecular orbitals establish bonds with and undergo mixing with the ligand molecular orbitals.

## Conclusions

Bis(terpyridine)manganese(III) exhibits Jahn–Teller distortion due to the presence of a single electron in the *e*_*g*_ set of the d orbitals of this high-spin d^4^ pseudo octahedral complex. The type of calculated Jahn–Teller distortion depends on the DFAs and is intimately related to the order of the frontier MOs produced by the DFA. For bis(terpyridine)manganese(III), compression Jahn–Teller structures were obtained by gas-phase optimizations, using the B3LYP, M06, and OLYP-D3 functionals. The PW6B95D3, MN15, and MN15-D3 functionals gave elongation Jahn–Teller structures in the gas phase. All solvent-phase optimization converged to elongation Jahn–Teller structures. Free energies show that elongation Jahn–Teller structures are generally preferred. Due to the spatially constrained nature of the terpyridine ligand, the central Mn-N bonds will always be shorter than the Mn-N terminal bonds, making it more difficult to distinguish between compression and elongation Jahn–Teller structures for bis(terpyridine)manganese(III). When obtaining an optimized bis(terpyridine)manganese(III) Jahn–Teller distorted structure, the following two rules can be used to distinguish between a compression and elongation Jahn–Teller geometry for bis(terpyridine)manganese(III):

(i) If the length of (Mn(III)-N_t2_)_average_ is nearer to (Mn(III)-N_t1_)_average_ than to (Mn(III)-N_c_)_average_, then it is a compression Jahn–Teller geometry (and vice versa for elongation Jahn–Teller).

(ii) The character of the LUMO is ${d}_{{x}^{2}-{y}^{2}}$ and ${d}_{{z}^{2}}$ respectively for elongation (*z*-direction along the longest terminal bonds) and compression (*z*-direction along the central bonds) Jahn–Teller geometry.

In bis(terpyridine)manganese(III), the character of the singly occupied *e*_*g*_ molecular orbital, which engages in an anti-bonding interaction with the nitrogen-p molecular orbitals, determines the specific manifestation of Jahn–Teller distortion. Concurrently, the low energy occupied bonding *t*_2g_ molecular orbitals establish bonds with and undergo mixing with the ligand molecular orbitals.

The OLYP-D3 functional is recommended for conducting calculations on bis(terpyridine)manganese(III) and related compounds for several reasons:

(i) OLYP-D3 consistently generated calculated metal–ligand bonds that were slightly longer than those observed in experiments, aligning with the required behavior.

(ii) OLYP-D3 provided a realistic electronic structure for bis(terpyridine)manganese(III), where the alpha *e*_*g*_ MOs were identified as the HOMO and LUMO. This aligns with expectations based on experimental electrochemical studies.

(iii) OLYP-D3 accurately reproduced the experimental *z*-in geometry for the only structurally known bis(terpyridine)manganese(III) compound, adding confidence in its reliability for these types of calculations.

### Supplementary Information

Below is the link to the electronic supplementary material.

Supplementary file1 (PDF 1410 KB)

## Data Availability

Optimized coordinates of the DFT calculations are in the supporting information. The datasets generated during and/or analysed during the current study are available from the corresponding author on reasonable request.
