# Supplementary material for: Effect of density functional approximations on the calculated Jahn–Teller distortion in bis(terpyridine)manganese(III) and related compounds
Source: J Mol Model. 2024 Jan 2;30(1):20. doi: 10.1007/s00894-023-05812-0 (PMC10761540; doi:10.1007/s00894-023-05812-0)
Supplement: Supplementary file 1 — Supplementary file1 (PDF 1410 KB) [file 894_2023_5812_MOESM1_ESM.pdf]

Effect of Density-Functional Approximations on the Calculated Jahn-Teller Distortion in Bis(terpyridine)manganese(III) and Related Compounds

Jeanet Conradie[a,b]*

a Department of Chemistry, University of the Free State, P.O. Box 339, Bloemfontein, 9300, South Africa

b UiT - The Arctic University of Norway, N-9037 Tromsø, Norway

Supporting information

Table of Contents

Table of Contents 1
1. Figures 3
2. Tables 5
3. OLYP-D3 Optimized Cartesian coordinates (Å) 10
Bis(terpyridine)manganese(II) q = 2, S = 5/2 (gas phase OLYP-D3) 10
Bis(terpyridine)manganese(III) q = 3, S = 2 (gas phase OLYP-D3) 11
Bis(terpyridine)manganese(II) q = 2, S = 5/2 (solvent phase OLYP-D3) 12
Bis(terpyridine)manganese(III) q = 3, S = 2 (solvent phase OLYP-D3) 13
Bis(4′-(4-methylphenyl)-2,2′:6′,2′′-terpyridine)manganese(II) q = 2, S = 5/2 (gas phase OLYP-D3) 15
Bis(4′-(4-methylphenyl)-2,2′:6′,2′′-terpyridine)manganese(III) q = 3, S = 2 (gas phase OLYP-D3) 16
Bis(4′-(4-methylphenyl)-2,2′:6′,2′′-terpyridine)manganese(II) q = 2, S = 5/2 (solvent phase OLYP-D3) 18
Bis(4′-(4-methylphenyl)-2,2′:6′,2′′-terpyridine)manganese(III) q = 3, S = 2 (solvent phase OLYP-D3) 20
4. PW6B95D3 Optimized Cartesian coordinates (Å) 22
Bis(terpyridine)manganese(II) q = 2, S = 5/2 (gas phase PW6B95D3) 22

Bis(terpyridine)manganese(III) q = 3, S = 2 (gas phase PW6B95D3) 23
Bis(4′-(4-methylphenyl)-2,2′:6′,2′′-terpyridine)manganese(II) q = 2, S = 5/2 (gas phase PW6B95D3) 24
Bis(4′-(4-methylphenyl)-2,2′:6′,2′′-terpyridine)manganese(III) q = 3, S = 2 (gas phase PW6B95D3) 26

1. Figures

	(a)	Compression	(b)	Elongation	(c)	Elongation
	MO no	MO fig and energy (contour 0.06)	MO no	MO fig and energy (contour 0.06)		MO fig ADF (contour 0.03)
HOMO -36	116 α	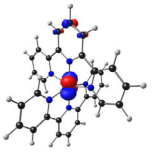 -17.06 eV	116 α	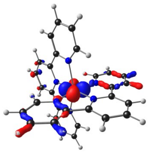 -17.17 eV	B2 28 α	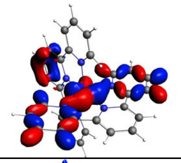
HOMO - 35/35/ 34	117 α	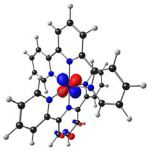 -17.06 eV	117 α	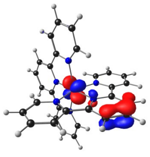 -17.01 eV	B1 28 α	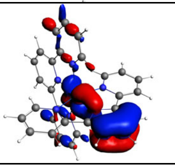
HOMO - 30/32/ 33	120 α	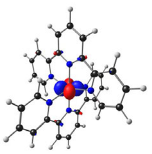 -16.71 eV	119 α	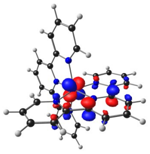 -16.84 eV	A2 3 α	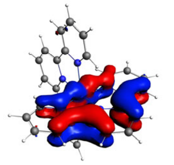
HOMO -6	131 α	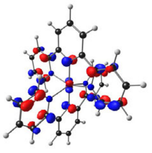 -15.21 eV	129 β	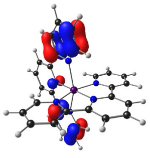 -15.13 eV	B2 31 β	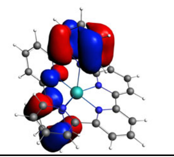
HOMO -5	132 α	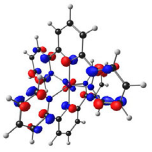 -15.21 eV	132 α	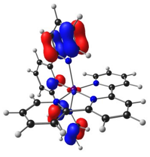 -15.08 eV	B2 32 α	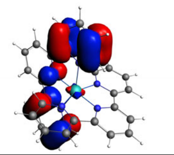
HOMO -4	133 α	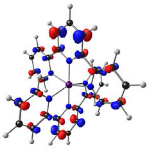 -14.59 eV	130 β	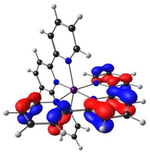 -14.78 eV	A2 7 β	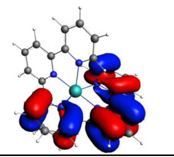
HOMO -3	130 β	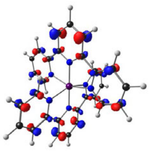 -14.56 eV	133 α	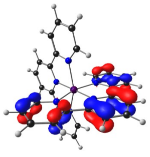 -14.78 eV	A2 8 α	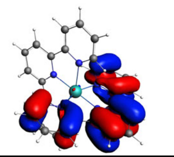
HOMO -2	131 β	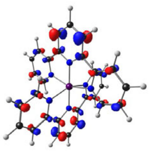 -14.56 eV	134 α	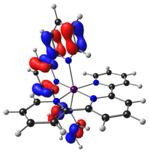 -14.42 eV	A2 9 α	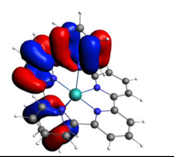
HOMO -1	134 α	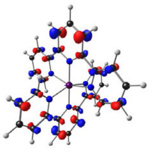 -14.56 eV	131 β	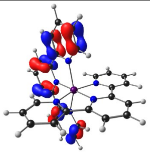 -14.42 eV	A2 8 β	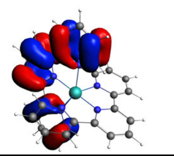
HOMO	135 α	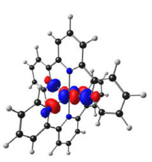 -13.61 eV	135 α	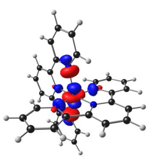 -13.63 eV	A1 62 α	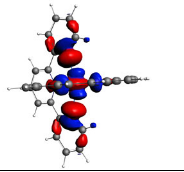

LUMO	136 α	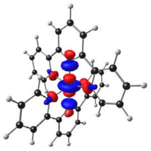 -13.14 eV	136 α	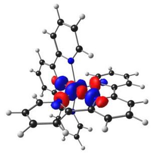 -13.09 eV	A1 63 α	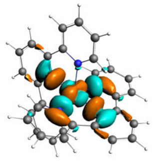
LUMO +1	132 β	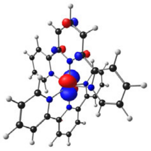 -12.60 eV	132 β	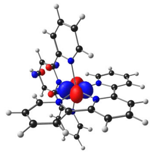 -12.65 eV	B2 32 β	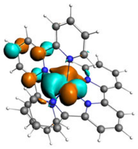
LUMO +2	133 β	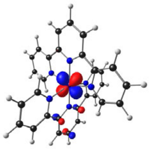 -12.60 eV	133 β	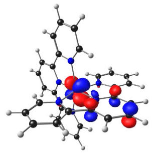 -12.57 eV	B1 32 β	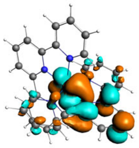
LUMO +3	134 β	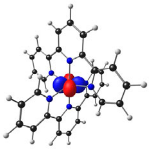 -12.14 eV	134 β	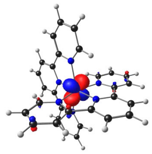 -12.22 eV	A2 9 β	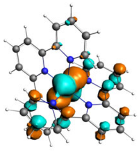
LUMO +12	139 β	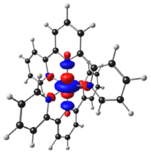 -10.97 eV	139 β	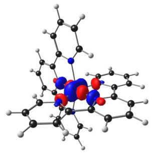 -10.94 eV	A1 62 β	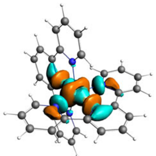
LUMO +13	140 β	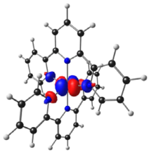 -10.59 eV	140 β	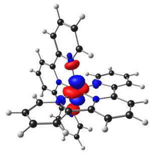 -10.64 eV	A1 62 β	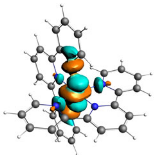

Figure S 1: Selected OLYP-D3 calculated MOs of the elongation and compression geometries of bis(terpyridine)manganese(III). (a) and (b) with 6-311G(d,p) basis set and contour 0.06 eÅ^{-3}. (c) with TZ2P basis set and contour 0.03 eÅ^{-3}.

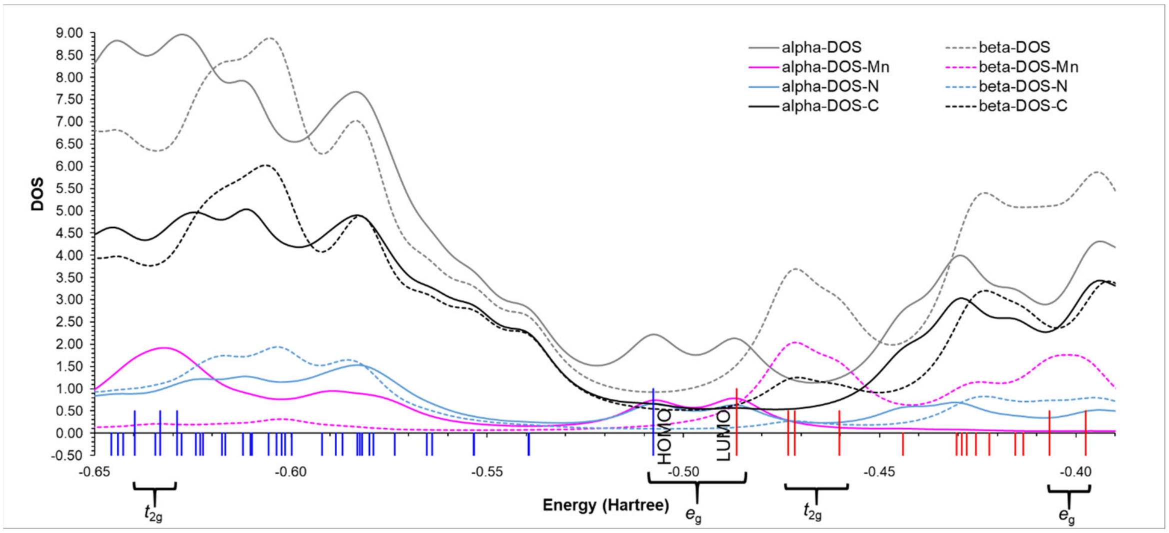

Figure S 2: Total and partial density of states of OLYP-D3 calculated elongation geometry of bis(terpyridine)manganese(III). At the bottom the energies of occupied (blue) and unoccupied (red) MOs are shown.

2. Tables

Table S 1. Atomic contributions to the indicated molecular orbitals for the OLYP-D3 elongated bis(terpyridine)manganese(III)

		No	No Energy (eV)	Character as fraction of 1 electron (only fraction above 0.05 i.e. a % above 5% indicated, except for e_g and t_{2g} MOs more detail in green font is given).																							
**HOMO-36**	α	116	-17.17	**Mn1-d**	0.5332	C8-p	0.041	C11-p	0.0309	C9-p	0.0308	N6-p	0.0235	N4-p	0.0222	C32-p	0.0205										
**HOMO-35**	α	117	-17.01	**Mn1-d**	0.4687	C37-p	0.1198	C35-p	0.0792	C36-p	0.0757	N7-p	0.0466														
HOMO-34	β	115	-16.98	N4-p	0.0924	N6-p	0.0845	**Mn1-d**	0.0837	C16-p	0.0524	C18-p	0.051														
HOMO-33	α	118	-16.84	**Mn1-d**	0.1631	C12-p	0.0538	C23-p	0.0531	C31-p	0.0407	C26-p	0.0405	C36-p	0.0376	C34-p	0.0352	C25-p	0.0343	N6-p	0.032	N4-p	0.0314	C35-p	0.0312		
**HOMO-32**	α	119	-16.84	**Mn1-d**	0.3786	C33-p	0.0736	C30-p	0.0719																		
HOMO-31	β	116	-16.71	N2-p	0.1848	N7-p	0.0786	C15-p	0.0609	C13-p	0.0607	C11-p	0.0566	C9-p	0.0564	H38-s	0.0503										
HOMO-30	α	120	-16.71	N2-p	0.1782	N7-p	0.1193	C15-p	0.0533	C13-p	0.0527	C11-p	0.0524	C9-p	0.0519												
HOMO-29	β	117	-16.71	C33-p	0.1766	C30-p	0.1764	C18-p	0.084	C24-p	0.0829	C36-p	0.0805	C35-p	0.0768	C16-p	0.0556	C27-p	0.0543	C26-p	0.0542	C31-p	0.0529				
HOMO-28	α	121	-16.65	**Mn1-d**	0.1668	C30-p	0.101	C33-p	0.1005	C16-p	0.0932	C27-p	0.0913	C18-p	0.0656	C24-p	0.0646	C21-p	0.0597	C32-p	0.0588						
HOMO-27	β	118	-16.52	C37-p	0.3369	N7-p	0.1838	C35-p	0.1832	C36-p	0.1791																
HOMO-26	β	119	-16.52	N6-p	0.1738	N4-p	0.1654																				
HOMO-25	α	122	-16.38	C8-p	0.1204	N6-p	0.0993	N4-p	0.096	N2-p	0.0842	C11-p	0.0678	C9-p	0.066												
HOMO-24	α	123	-16.35	C37-p	0.1373	**Mn1-d**	0.0894	N7-p	0.0799	C35-p	0.0676	C36-p	0.0647														
HOMO-23	β	120	-16.33	C13-p	0.1746	C15-p	0.1746	C9-p	0.0825	C11-p	0.0814	C22-p	0.0793	C29-p	0.079	C17-p	0.055	C28-p	0.0546								
HOMO-22	α	124	-16.33	C13-p	0.1695	C15-p	0.1695	C22-p	0.0813	C29-p	0.0811	C9-p	0.0773	C11-p	0.0761	C17-p	0.0702	C28-p	0.0699								
HOMO-21	β	121	-16.25	C8-p	0.3488	N2-p	0.1969	C11-p	0.1826	C9-p	0.1816																
HOMO-20	β	122	-16.16	N3-p	0.1507	N5-p	0.1286	**Mn1-d**	0.1119																		
HOMO-19	β	123	-16.14	C21-p	0.1816	C32-p	0.1247	C25-p	0.1186	N4-p	0.1085	C34-p	0.0793	C16-p	0.0742	N6-p	0.0727	C27-p	0.0533								
HOMO-18	β	124	-16.11	C32-p	0.1449	C34-p	0.1084	N6-p	0.0952	C21-p	0.0894	C25-p	0.0703	N4-p	0.0614	N5-p	0.0564										
HOMO-17	α	125	-16.05	C37-p	0.1081	C21-p	0.1054	C25-p	0.1022	C32-p	0.101	C34-p	0.0985	N4-p	0.0868	N6-p	0.0833	**Mn1-d**	0.061								
HOMO-16	α	126	-15.86	C8-p	0.2287	**Mn1-d**	0.2132	N2-p	0.0933	C9-p	0.0933	C11-p	0.0931														
HOMO-15	α	127	-15.76	**Mn1-d**	0.1721	C32-p	0.1248	C21-p	0.1155	N6-p	0.0911	C34-p	0.0872	N4-p	0.0842	C25-p	0.0814										

HOMO-14	β	125	-15.73	N5-p	0.1636	N3-p	0.1613																				
HOMO-13	α	128	-15.62	C27-p	0.0901	N5-p	0.0899	C16-p	0.0874	N3-p	0.0872	Mn1-d	0.0697	C26-p	0.0696	C31-p	0.0689										
HOMO-12	β	126	-15.59	C12-p	0.1734	C23-p	0.1393	N3-p	0.1247	C10-p	0.1172	N5-p	0.1005	C19-p	0.0949	C17-p	0.0531										
HOMO-11	α	129	-15.59	C12-p	0.1784	C23-p	0.1599	N3-p	0.1151	N5-p	0.1024	C10-p	0.1005	C19-p	0.089	C17-p	0.0668	C28-p	0.0609								
HOMO-10	β	127	-15.56	C23-p	0.1844	C12-p	0.1496	N5-p	0.1292	C19-p	0.1221	N3-p	0.1048	C10-p	0.099	C28-p	0.0566										
HOMO-9	α	130	-15.51	Mn1-d	0.1673	C23-p	0.1441	C12-p	0.1294	N5-p	0.1014	C19-p	0.0994	N3-p	0.0917	C10-p	0.0905										
HOMO-8	β	128	-15.48	C26-p	0.1351	C31-p	0.1342	C16-p	0.1278	C27-p	0.1271	C18-p	0.1129	C24-p	0.1118	N7-p	0.0735	C25-p	0.0628	C34-p	0.061						
HOMO-7	α	131	-15.35	Mn1-d	0.144	C18-p	0.0811	N7-p	0.0767	C24-p	0.0765	C25-p	0.0646	N5-p	0.061	N3-p	0.0608	C34-p	0.0601	C26-p	0.0576	C31-p	0.0572	C16-p	0.0504	C27-p	0.0503
HOMO-6	β	129	-15.13	C14-p	0.1347	C20-p	0.1341	C17-p	0.1234	C28-p	0.1231	C22-p	0.1155	C29-p	0.1149	N2-p	0.0689	C10-p	0.0676	C19-p	0.0665						
HOMO-5	α	132	-15.08	C14-p	0.1243	C20-p	0.1237	C22-p	0.1173	C29-p	0.1167	C17-p	0.113	C28-p	0.1124	N2-p	0.0863	C10-p	0.076	C19-p	0.0749						
HOMO-4	β	130	-14.78	C35-p	0.125	C36-p	0.1248	C27-p	0.0832	C16-p	0.0811	C34-p	0.0767	C25-p	0.0756	C24-p	0.0715	C18-p	0.0699	C30-p	0.0661	C33-p	0.0657	C31-p	0.0582	C26-p	0.0561
HOMO-3	α	133	-14.78	C35-p	0.1258	C36-p	0.1254	C27-p	0.0869	C16-p	0.0846	C34-p	0.0693	C25-p	0.0677	C30-p	0.067	C33-p	0.067	C24-p	0.0634	C31-p	0.0628	C18-p	0.0614	C26-p	0.0607
HOMO-2	α	134	-14.42	C11-p	0.1218	C9-p	0.1217	C28-p	0.0876	C17-p	0.0864	C19-p	0.0743	C10-p	0.0736	C29-p	0.0681	C22-p	0.067	C20-p	0.0639	C14-p	0.0627	C13-p	0.0623	C15-p	0.062
HOMO-1	β	131	-14.42	C11-p	0.123	C9-p	0.1228	C28-p	0.0855	C17-p	0.0843	C19-p	0.0765	C10-p	0.0758	C29-p	0.071	C22-p	0.07	C20-p	0.062	C13-p	0.0615	C15-p	0.0612	C14-p	0.0609
HOMO	α	135	-13.63	Mn1-d	0.4606	N3-p	0.1131	N5-p	0.112	N6-p	0.04	N4-p	0.0379	N3-s	0.0242	N5-s	0.024	N2-p	0.0209	N6-p	0.04						
LUMO	α	136	-13.09	Mn1-d	0.5127	N7-p	0.0956	N2-p	0.0754	N4-p	0.0594	N6-p	0.0581														
LUMO+1	β	132	-12.65	Mn1-d	0.7409	C8-p	0.0449	C15-p	0.0386	C13-p	0.0386																
LUMO+2	β	133	-12.57	Mn1-d	0.5497	C37-p	0.0826	C33-p	0.0694	C30-p	0.0693																
LUMO+3	β	134	-12.22	Mn1-d	0.7065	C21-p	0.0287	C32-p	0.0268	C26-p	0.0268	C31-p	0.0251	C18-p	0.024	C24-p	0.0225	C21-p	0.0287								
LUMO+4	α	137	-11.78	N7-p	0.169	C37-p	0.1352	C30-p	0.0954	C33-p	0.0937	N4-p	0.0553	N6-p	0.0533												
LUMO+5	α	138	-11.43	N2-p	0.1767	C8-p	0.1506	C13-p	0.1035	C15-p	0.1026	N3-p	0.0511	N5-p	0.0502												
LUMO+6	β	135	-11.40	Mn1-d	0.3389	N7-p	0.1229	C37-p	0.0647																		
LUMO+7	α	139	-11.37	C35-p	0.1941	C36-p	0.1924	C33-p	0.0617	C27-p	0.0593	C30-p	0.0589	C16-p	0.0578	C31-p	0.0568	C26-p	0.0559								
LUMO+8	β	136	-11.29	C35-p	0.1822	C36-p	0.1785	Mn1-d	0.0731	C33-p	0.0695	C27-p	0.0642	C30-p	0.0639	C16-p	0.0598										
LUMO+9	β	137	-11.24	N2-p	0.1611	Mn1-d	0.1498	C8-p	0.1178	C13-p	0.0749	C15-p	0.0738														
LUMO+10	α	140	-11.02	C9-p	0.196	C11-p	0.1952	C15-p	0.0681	C13-p	0.0669	C28-p	0.059	C17-p	0.0586	C20-p	0.0508	C14-p	0.0505								

LUMO +11	β	138	-10.97	C9-p	0.1924	C11-p	0.1914	C15-p	0.0738	C13-p	0.0724	C28-p	0.0611	C17-p	0.0603												
**LUMO +12**	β	139	-10.94	**Mn1-d**	0.7182	N7-p	0.0482	N2-p	0.0384	N4-p	0.0271	N6-p	0.0263														
**LUMO +13**	β	140	-10.64	**Mn1-d**	0.7812	N3-p	0.0359	N5-p	0.0356																		

Table S 2. Atomic contributions to the indicated molecular orbitals for the OLYP-D3 compressed D_{2d} bis(terpyridine)manganese(III)

		No	Energy (eV)	Character as fraction of 1 electron (only fraction above 0.05 i.e. a % above 5% indicated, except for e_g and t_{2g} MOs more detail in green font is given).																							
**HOMO -36**	α	116	-17.06	**Mn1-d**	0.535	C37-p	0.0668	C36-p	0.0465	C35-p	0.0465	N7-p	0.0175	C12-p	0.0147	C23-p	0.0147	C28-p	0.0134	C17-p	0.0134	N3-p	0.0133	N5-p	0.0133	C29-p	0.0133
**HOMO -35**	α	117	-17.06	**Mn1-d**	0.535	C8-p	0.0668	C9-p	0.0465	N2-p	0.0175	C32-p	0.0147	C21-p	0.0147	C16-p	0.0134	C27-p	0.0134	N6-p	0.0133	N4-p	0.0133	C18-p	0.0133	C24-p	0.0133
HOMO -34	α	118	-17.01	Mn1-s	0.066	Mn1-d	0.0496	C32-p	0.0319	C21-p	0.0319	C12-p	0.0319	C23-p	0.0319	H49-s	0.029	H47-s	0.029	H41-s	0.029	H55-s	0.029	C29-p	0.0282	C22-p	0.0282
HOMO -33	β	115	-16.84	N7-p	0.134	N2-p	0.1344																				
HOMO -32	α	119	-16.79	N7-p	0.155	N2-p	0.1549																				
HOMO -31	β	116	-16.74	**Mn1-d**	0.066	N5-p	0.0518	N3-p	0.0518	N6-p	0.0518	N4-p	0.0518														
**HOMO -30**	α	120	-16.71	**Mn1-d**	0.515	C34-p	0.0255	C10-p	0.0255	C19-p	0.0255	C25-p	0.0255	C30-p	0.0253	C15-p	0.0253	C13-p	0.0253	C33-p	0.0253	C31-p	0.0204	C20-p	0.0204	C14-p	0.0204
HOMO -29	α	121	-16.54	C33-p	0.087	C13-p	0.0871	C30-p	0.0871	C15-p	0.0871																
HOMO -28	β	117	-16.52	C30-p	0.086	C33-p	0.0861	C13-p	0.0861	C15-p	0.0861																
HOMO -27	β	118	-16.46	C13-p	0.092	C15-p	0.0924	C30-p	0.0924	C33-p	0.0924																
HOMO -26	α	122	-16.44	**Mn1-d**	0.091	C13-p	0.0687	C15-p	0.0687	C30-p	0.0687	C33-p	0.0687														
HOMO -25	β	119	-16.38	C8-p	0.251	N2-p	0.1424	C11-p	0.1333	C9-p	0.1333	C37-p	0.0892	N7-p	0.0507												
HOMO -24	β	120	-16.38	C37-p	0.251	N7-p	0.1424	C36-p	0.1333	C35-p	0.1333	C8-p	0.0892	N2-p	0.0507												
HOMO -23	β	121	-16.35	**Mn1-d**	0.125	N6-p	0.0722	N3-p	0.0722	N5-p	0.0722	N4-p	0.0722														
HOMO -22	α	123	-16.35	C8-p	0.128	N2-p	0.0829	C9-p	0.0655	C11-p	0.0655	N6-p	0.0569	N4-p	0.0569												
HOMO -21	α	124	-16.35	C37-p	0.128	N7-p	0.0829	C35-p	0.0655	C36-p	0.0655	N5-p	0.0569	N3-p	0.0569												
HOMO -20	β	122	-16.19	N3-p	0.106	N5-p	0.1058	N4-p	0.0736	N6-p	0.0736																
HOMO -19	β	123	-16.19	N4-p	0.106	N6-p	0.1058	N5-p	0.0736	N3-p	0.0736																
HOMO -18	β	124	-16.00	C12-p	0.090	C23-p	0.09	C32-p	0.09	C21-p	0.09	C10-p	0.0583	C25-p	0.0583	C19-p	0.0583	C34-p	0.0583	N3-p	0.0501	N5-p	0.0501	N6-p	0.0501	N4-p	0.0501
HOMO -17	α	125	-15.89	**Mn1-d**	0.129	C8-p	0.1286	C10-p	0.0589	C19-p	0.0589	C12-p	0.051	C23-p	0.051	N3-p	0.0507	N5-p	0.0507								

HOMO -16	α	126	-15.89	**Mn1-d**	0.129	C37-p	0.1286	C34-p	0.0589	C25-p	0.0589	C32-p	0.051	C21-p	0.051	N6-p	0.0507	N4-p	0.0507								
HOMO -15	β	125	-15.73	C12-p	0.080	C23-p	0.0801	C32-p	0.0726	C21-p	0.0726	N3-p	0.0678	N5-p	0.0678	N6-p	0.0636	N4-p	0.0636	C10-p	0.0549	C19-p	0.0549				
HOMO -14	β	126	-15.73	C21-p	0.080	C32-p	0.0801	C23-p	0.0726	C12-p	0.0726	N4-p	0.0678	N6-p	0.0678	N5-p	0.0636	N3-p	0.0636	C25-p	0.0549	C34-p	0.0549				
HOMO -13	α	127	-15.70	C23-p	0.087	C32-p	0.0865	C21-p	0.0865	C12-p	0.0865	N5-p	0.0651	N6-p	0.0651	N4-p	0.0651	N3-p	0.0651	C19-p	0.0584	C34-p	0.0584	C25-p	0.0584	C10-p	0.0584
HOMO -12	β	127	-15.67	C21-p	0.086	C23-p	0.0861	C12-p	0.0861	C32-p	0.0861	N4-p	0.0655	N5-p	0.0655	N3-p	0.0655	N6-p	0.0655	C34-p	0.0584	C10-p	0.0584	C19-p	0.0584	C25-p	0.0584
HOMO -11	α	128	-15.65	**Mn1-d**	0.109	C21-p	0.1069	C32-p	0.1069	N5-p	0.075	N3-p	0.075	C16-p	0.0614	C27-p	0.0614	N4-p	0.0583	N6-p	0.0583						
HOMO -10	α	129	-15.65	**Mn1-d**	0.109	C12-p	0.1069	C23-p	0.1069	N6-p	0.075	N4-p	0.075	C17-p	0.0614	C28-p	0.0614	N3-p	0.0583	N5-p	0.0583						
HOMO -9	α	130	-15.54	**Mn1-d**	0.316	C23-p	0.0592	C21-p	0.0592	C32-p	0.0592	C12-p	0.0592														
HOMO -8	β	128	-15.29	C31-p	0.134	C26-p	0.1344	C27-p	0.1239	C16-p	0.1239	C18-p	0.1135	C24-p	0.1135	N7-p	0.0696	C25-p	0.0646	C34-p	0.0646						
HOMO -7	β	129	-15.29	C14-p	0.134	C20-p	0.1344	C17-p	0.1239	C28-p	0.1239	C22-p	0.1135	C29-p	0.1135	N2-p	0.0696	C10-p	0.0646	C19-p	0.0646						
HOMO -6	α	131	-15.21	C26-p	0.109	C31-p	0.1091	C18-p	0.1077	C24-p	0.1077	C16-p	0.0984	C27-p	0.0984	N7-p	0.0895	C25-p	0.0718	C34-p	0.0718						
HOMO -5	α	132	-15.21	C14-p	0.109	C20-p	0.1091	C22-p	0.1077	C29-p	0.1077	C17-p	0.0984	C28-p	0.0984	N2-p	0.0895	C10-p	0.0718	C19-p	0.0718						
HOMO -4	α	133	-14.59	C11-p	0.064	C9-p	0.0638	C36-p	0.0638	C35-p	0.0638																
HOMO -3	β	130	-14.56	C36-p	0.059	C35-p	0.0593	C11-p	0.0593	C9-p	0.0593																
HOMO -2	β	131	-14.56	C9-p	0.059	C11-p	0.0588	C36-p	0.0588	C35-p	0.0588																
HOMO -1	α	134	-14.56	C36-p	0.065	C35-p	0.0648	C11-p	0.0648	C9-p	0.0648																
HOMO	α	135	-13.61	**Mn1-d**	0.473	N5-p	0.0776	N3-p	0.0776	N4-p	0.0776	N6-p	0.0776	N6-s	0.0183	N3-s	0.0183	N5-s	0.0183	N4-s	0.0183						
LUMO	α	136	-13.14	**Mn1-d**	0.507	N7-p	0.0923	N2-p	0.0923	N4-p	0.0295	N6-p	0.0295	N5-p	0.0295	N3-p	0.0295	N2-s	0.0184	N7-s	0.0184						
LUMO +1	β	132	-12.60	**Mn1-d**	0.655	C37-p	0.0595	C33-p	0.0509	C30-p	0.0509	N7-p	0.0175														
LUMO +2	β	133	-12.60	**Mn1-d**	0.655	C8-p	0.0595	C13-p	0.0509	C15-p	0.0509	N7-p	0.0175														
LUMO +3	β	134	-12.14	**Mn1-d**	0.727	C21-p	0.0181	C12-p	0.0181	C23-p	0.0181	C32-p	0.0181	C14-p	0.017	C26-p	0.017	C31-p	0.017	C20-p	0.017	C18-p	0.0157	C29-p	0.0157	C24-p	0.0157
LUMO +4	α	137	-11.62	N2-p	0.162	C8-p	0.1352	C13-p	0.0939	C15-p	0.0939																
LUMO +5	α	138	-11.62	N7-p	0.162	C37-p	0.1352	C33-p	0.0939	C30-p	0.0939																
LUMO +6	β	135	-11.32	**Mn1-d**	0.237	N2-p	0.1429	C8-p	0.0921	C13-p	0.0569	C15-p	0.0569														
LUMO +7	β	136	-11.32	**Mn1-d**	0.237	N7-p	0.1429	C37-p	0.0921	C33-p	0.0569	C30-p	0.0569														
LUMO +8	α	139	-11.21	C9-p	0.097	C11-p	0.0971	C36-p	0.0971	C35-p	0.0971																

LUMO +9	α	140	-11.18	C36-p	0.100	C35-p	0.0998	C11-p	0.0998	C9-p	0.0998																
LUMO +10	β	137	-11.16	C36-p	0.099	C11-p	0.0986	C9-p	0.0986	C35-p	0.0986																
LUMO +11	β	138	-11.08	**Mn1-d**	0.099	C9-p	0.0927	C11-p	0.0927	C36-p	0.0927	C35-p	0.0927														
**LUMO +12**	β	139	-10.97	**Mn1-d**	0.718	N2-p	0.0451	N7-p	0.0451	N5-p	0.0141	N3-p	0.0141	N4-p	0.0141	N6-p	0.0141	Mn1-s	0.012	N2-s	0.0109	N7-s	0.0109				
**LUMO +13**	β	140	-10.59	**Mn1-d**	0.791	N4-p	0.0253	N6-p	0.0253	N3-p	0.0253	N5-p	0.0253														

3. OLYP-D3 Optimized Cartesian coordinates (Å)

Bis(terpyridine)manganese(II) q = 2, S = 5/2 (gas phase OLYP-D3)

Mn	0.000000000	0.000000000	0.000000000
N	-2.168517000	0.000000000	0.697807000
C	-2.392227000	0.000000000	2.035849000
C	-3.693861000	0.000000000	2.553053000
C	-4.779576000	0.000000000	1.677629000
C	-4.542835000	0.000000000	0.303426000
C	-3.220303000	0.000000000	-0.136636000
N	0.000000000	0.000000000	2.227006000
C	-1.175190000	0.000000000	2.892084000
C	-1.206113000	0.000000000	4.293242000
C	0.000000000	0.000000000	4.991445000
C	1.206113000	0.000000000	4.293242000
C	1.175190000	0.000000000	2.892084000
N	2.168517000	0.000000000	0.697807000
C	2.392227000	0.000000000	2.035849000
C	3.693861000	0.000000000	2.553053000
C	4.779576000	0.000000000	1.677629000
C	4.542835000	0.000000000	0.303426000
C	3.220303000	0.000000000	-0.136636000
N	0.000000000	2.168517000	-0.697807000
C	0.000000000	2.392227000	-2.035849000
C	0.000000000	3.693861000	-2.553053000
C	0.000000000	4.779576000	-1.677629000
C	0.000000000	4.542835000	-0.303426000
C	0.000000000	3.220303000	0.136636000
N	0.000000000	0.000000000	-2.227006000
C	0.000000000	1.175190000	-2.892084000
C	0.000000000	1.206113000	-4.293242000
C	0.000000000	0.000000000	-4.991445000
C	0.000000000	-1.206113000	-4.293242000
C	0.000000000	-1.175190000	-2.892084000
N	0.000000000	-2.168517000	-0.697807000
C	0.000000000	-2.392227000	-2.035849000
C	0.000000000	-3.693861000	-2.553053000
C	0.000000000	-4.779576000	-1.677629000
C	0.000000000	-4.542835000	-0.303426000
C	0.000000000	-3.220303000	0.136636000
H	-3.870971000	0.000000000	3.622262000
H	-5.794358000	0.000000000	2.066542000
H	-5.358469000	0.000000000	-0.413306000
H	-2.989144000	0.000000000	-1.199599000
H	-2.142673000	0.000000000	4.837833000

H	0.000000000	0.000000000	6.078183000
H	2.142673000	0.000000000	4.837833000
H	3.870971000	0.000000000	3.622262000
H	5.794358000	0.000000000	2.066542000
H	5.358469000	0.000000000	-0.413306000
H	2.989144000	0.000000000	-1.199599000
H	0.000000000	3.870971000	-3.622262000
H	0.000000000	5.794358000	-2.066542000
H	0.000000000	5.358469000	0.413306000
H	0.000000000	2.989144000	1.199599000
H	0.000000000	2.142673000	-4.837833000
H	0.000000000	0.000000000	-6.078183000
H	0.000000000	-2.142673000	-4.837833000
H	0.000000000	-3.870971000	-3.622262000
H	0.000000000	-5.794358000	-2.066542000
H	0.000000000	-5.358469000	0.413306000
H	0.000000000	-2.989144000	1.199599000

Bis(terpyridine)manganese(III) q = 3, S = 2 (gas phase OLYP-D3)

Mn	0.000000000	0.000000000	0.000000000
N	0.000000000	0.000000000	2.001485000
N	0.000000000	2.125990000	0.477419000
N	-2.125990000	0.000000000	-0.477419000
N	0.000000000	-2.125990000	0.477419000
N	2.125990000	0.000000000	-0.477419000
N	0.000000000	0.000000000	-2.001485000
C	0.000000000	0.000000000	4.765735000
C	0.000000000	1.207247000	4.067278000
C	0.000000000	3.703987000	2.292441000
C	0.000000000	-1.207247000	4.067278000
C	0.000000000	4.762759000	1.381276000
C	0.000000000	1.192037000	2.669351000
C	0.000000000	2.390561000	1.813547000
C	0.000000000	-1.192037000	2.669351000
C	-4.483038000	0.000000000	-0.014634000
C	0.000000000	4.483038000	0.014634000
C	-3.149243000	0.000000000	0.394183000
C	0.000000000	-3.703987000	2.292441000
C	0.000000000	-2.390561000	1.813547000
C	-4.762759000	0.000000000	-1.381276000
C	0.000000000	3.149243000	-0.394183000
C	0.000000000	-4.762759000	1.381276000
C	3.149243000	0.000000000	0.394183000
C	-3.703987000	0.000000000	-2.292441000
C	-2.390561000	0.000000000	-1.813547000
C	4.483038000	0.000000000	-0.014634000
C	0.000000000	-4.483038000	0.014634000
C	0.000000000	-3.149243000	-0.394183000
C	-1.192037000	0.000000000	-2.669351000
C	2.390561000	0.000000000	-1.813547000

C	4.762759000	0.000000000	-1.381276000
C	1.192037000	0.000000000	-2.669351000
C	3.703987000	0.000000000	-2.292441000
C	-1.207247000	0.000000000	-4.067278000
C	1.207247000	0.000000000	-4.067278000
C	0.000000000	0.000000000	-4.765735000
H	0.000000000	0.000000000	5.852912000
H	0.000000000	2.146506000	4.608148000
H	0.000000000	3.911530000	3.356770000
H	0.000000000	5.789650000	1.737747000
H	0.000000000	-2.146506000	4.608148000
H	-5.277027000	0.000000000	0.726455000
H	-2.891934000	0.000000000	1.449342000
H	0.000000000	-3.911530000	3.356770000
H	0.000000000	5.277027000	-0.726455000
H	-5.789650000	0.000000000	-1.737747000
H	2.891934000	0.000000000	1.449342000
H	0.000000000	-5.789650000	1.737747000
H	0.000000000	2.891934000	-1.449342000
H	5.277027000	0.000000000	0.726455000
H	-3.911530000	0.000000000	-3.356770000
H	0.000000000	-5.277027000	-0.726455000
H	0.000000000	-2.891934000	-1.449342000
H	5.789650000	0.000000000	-1.737747000
H	-2.146506000	0.000000000	-4.608148000
H	3.911530000	0.000000000	-3.356770000
H	2.146506000	0.000000000	-4.608148000
H	0.000000000	0.000000000	-5.852912000

Bis(terpyridine)manganese(II) q = 2, S = 5/2 (solvent phase OLYP-D3)

Mn	-0.000010000	0.041078000	-0.000001000
N	1.554098000	1.562728000	-0.700874000
C	1.720486000	1.699314000	-2.038600000
C	2.679584000	2.574037000	-2.564574000
C	3.471310000	3.322031000	-1.694552000
C	3.290062000	3.181238000	-0.319432000
C	2.319598000	2.287238000	0.129675000
N	0.024726000	0.011832000	-2.226616000
C	0.829869000	0.866334000	-2.889862000
C	0.800294000	0.941951000	-4.288498000
C	-0.076918000	0.114134000	-4.985210000
C	-0.899695000	-0.768370000	-4.289295000
C	-0.824396000	-0.798088000	-2.890636000
N	-1.516880000	-1.525729000	-0.702159000
C	-1.651478000	-1.694383000	-2.039726000
C	-2.518202000	-2.660520000	-2.566060000
C	-3.260724000	-3.457542000	-1.696171000
C	-3.122415000	-3.274121000	-0.321088000
C	-2.236277000	-2.296371000	0.127911000
N	-1.554107000	1.562730000	0.700885000

C	-1.720486000	1.699325000	2.038610000
C	-2.679574000	2.574059000	2.564585000
C	-3.471300000	3.322053000	1.694563000
C	-3.290064000	3.181248000	0.319442000
C	-2.319608000	2.287238000	-0.129666000
N	-0.024727000	0.011843000	2.226619000
C	-0.829869000	0.866343000	2.889869000
C	-0.800292000	0.941954000	4.288506000
C	0.076923000	0.114136000	4.985212000
C	0.899699000	-0.768365000	4.289292000
C	0.824397000	-0.798079000	2.890633000
N	1.516866000	-1.525722000	0.702151000
C	1.651477000	-1.694371000	2.039719000
C	2.518212000	-2.660500000	2.566048000
C	3.260731000	-3.457521000	1.696156000
C	3.122407000	-3.274108000	0.321073000
C	2.236259000	-2.296365000	-0.127921000
H	2.817743000	2.673332000	-3.634377000
H	4.219579000	4.003520000	-2.089575000
H	3.883225000	3.744554000	0.393956000
H	2.147191000	2.143364000	1.193224000
H	1.436019000	1.631713000	-4.829362000
H	-0.121434000	0.158916000	-6.069580000
H	-1.585397000	-1.408113000	-4.830605000
H	-2.615404000	-2.800071000	-3.635901000
H	-3.936039000	-4.211378000	-2.091223000
H	-3.681734000	-3.871000000	0.392367000
H	-2.094654000	-2.121247000	1.191391000
H	-2.817724000	2.673364000	3.634388000
H	-4.219561000	4.003550000	2.089586000
H	-3.883228000	3.744563000	-0.393945000
H	-2.147209000	2.143356000	-1.193214000
H	-1.436017000	1.631712000	4.829373000
H	0.121441000	0.158913000	6.069583000
H	1.585403000	-1.408108000	4.830599000
H	2.615426000	-2.800045000	3.635889000
H	3.936054000	-4.211351000	2.091205000
H	3.681723000	-3.870988000	-0.392384000
H	2.094625000	-2.121247000	-1.191401000

Bis(terpyridine)manganese(III) q = 3, S = 2 (solvent phase OLYP-D3)

Mn	0.016211000	0.030980000	0.077265000
N	-0.385610000	-0.759937000	-1.760721000
N	-0.625478000	1.797801000	-1.141903000
N	2.047234000	0.498969000	-0.141880000
N	0.395483000	-2.164519000	0.338890000
N	-1.843399000	-0.099704000	1.042033000
N	0.431070000	0.813157000	1.844020000
C	-0.890524000	-1.824709000	-4.261158000
C	-1.037649000	-0.458274000	-4.042273000

C	-1.320030000	2.475193000	-3.337696000
C	-0.490360000	-2.653771000	-3.217613000
C	-1.424336000	3.795882000	-2.902214000
C	-0.780885000	0.063395000	-2.772241000
C	-0.916936000	1.491854000	-2.429108000
C	-0.237402000	-2.099578000	-1.960567000
C	4.135750000	0.642174000	-1.301246000
C	-1.126383000	4.102337000	-1.575714000
C	2.788532000	0.292331000	-1.243256000
C	0.388704000	-4.271015000	-0.813601000
C	0.194858000	-2.886663000	-0.789881000
C	4.728879000	1.221700000	-0.181756000
C	-0.729739000	3.069769000	-0.727572000
C	0.795390000	-4.916540000	0.353604000
C	-2.970798000	-0.603051000	0.512970000
C	3.962031000	1.437656000	0.963502000
C	2.617524000	1.068368000	0.960171000
C	-4.164398000	-0.651375000	1.229888000
C	0.998425000	-4.167661000	1.511143000
C	0.786796000	-2.790673000	1.459133000
C	1.697165000	1.243810000	2.089735000
C	-1.852903000	0.383906000	2.318592000
C	-4.185656000	-0.161148000	2.533806000
C	-0.557799000	0.904509000	2.772886000
C	-3.016775000	0.363588000	3.086040000
C	2.010510000	1.798013000	3.331677000
C	-0.282598000	1.455485000	4.025139000
C	1.010304000	1.900932000	4.297787000
H	-1.087622000	-2.243147000	-5.243365000
H	-1.348531000	0.191086000	-4.850161000
H	-1.552323000	2.227470000	-4.366015000
H	-1.736312000	4.572229000	-3.594571000
H	-0.375919000	-3.717238000	-3.381413000
H	4.695311000	0.456720000	-2.211807000
H	2.293343000	-0.164133000	-2.092118000
H	0.228125000	-4.844204000	-1.718322000
H	-1.196625000	5.116216000	-1.195781000
H	5.777078000	1.504576000	-0.194428000
H	-2.915093000	-0.974217000	-0.503646000
H	0.950339000	-5.991400000	0.353471000
H	-0.490948000	3.262724000	0.313940000
H	-5.050218000	-1.067450000	0.762051000
H	4.407162000	1.887218000	1.842972000
H	1.314530000	-4.630273000	2.440323000
H	0.936071000	-2.172790000	2.339052000
H	-5.099598000	-0.184172000	3.119505000
H	3.014538000	2.145141000	3.541694000
H	-3.015992000	0.749969000	4.098062000
H	-1.060297000	1.536387000	4.774406000
H	1.239408000	2.331372000	5.267739000

Bis(4′-(4-methylphenyl)-2,2′:6′,2′′-terpyridine)manganese(II) q = 2, S = 5/2 (gas phase OLYP-D3)

Mn	-0.000002000	-0.000541000	-0.000548000
N	-0.696523000	1.533533000	-1.541172000
N	-2.205340000	-0.002803000	-0.002075000
N	-0.695492000	-1.535875000	1.539287000
N	0.697673000	-1.534883000	-1.540527000
N	2.205339000	0.000921000	0.000209000
N	0.694339000	1.534591000	1.539865000
C	0.135844000	2.275303000	-2.289192000
C	-0.307266000	3.203504000	-3.228877000
C	-1.682782000	3.365331000	-3.394928000
C	-2.554928000	2.600417000	-2.621889000
C	-2.035153000	1.685667000	-1.696595000
C	-2.885005000	0.826205000	-0.826321000
C	-4.279360000	0.847037000	-0.847938000
C	-5.019079000	-0.000400000	0.001281000
C	-4.278806000	-0.849140000	0.848683000
C	-2.884450000	-0.830725000	0.823680000
C	-2.034022000	-1.691236000	1.692360000
C	-2.553213000	-2.610053000	2.613941000
C	-1.680585000	-3.375196000	3.386212000
C	-0.305177000	-3.209614000	3.223015000
C	0.137344000	-2.277882000	2.286548000
C	-6.488081000	0.001078000	0.002690000
C	-7.215775000	-1.180938000	0.248109000
C	-8.605946000	-1.175986000	0.242577000
C	-9.331916000	0.005362000	0.010357000
C	-8.603702000	1.185032000	-0.222969000
C	-7.213479000	1.185488000	-0.237547000
C	-10.834267000	0.001466000	-0.014284000
C	-0.134142000	-2.277183000	-2.288632000
C	0.309663000	-3.206648000	-3.226741000
C	1.685302000	-3.369331000	-3.390930000
C	2.556876000	-2.603864000	-2.617794000
C	2.036413000	-1.687684000	-1.694302000
C	2.885624000	-0.827378000	-0.824235000
C	4.279996000	-0.846665000	-0.846356000
C	5.019079000	0.001616000	0.002572000
C	4.278171000	0.849535000	0.850241000
C	2.883829000	0.829559000	0.825758000
C	2.032760000	1.689213000	1.694660000
C	2.551266000	2.606515000	2.618133000
C	1.678067000	3.371105000	3.390307000
C	0.302779000	3.206464000	3.225159000
C	-0.139049000	2.276067000	2.287042000
C	6.488083000	0.001868000	0.003352000
C	7.214772000	-1.181758000	-0.237179000
C	8.604936000	-1.179701000	-0.223187000
C	9.331923000	0.000879000	0.009813000

C	8.604725000	1.181332000	0.242287000
C	7.214488000	1.184684000	0.248413000
C	10.834259000	0.005942000	-0.015540000
H	1.199079000	2.114048000	-2.124483000
H	0.406789000	3.778913000	-3.810224000
H	-2.074602000	4.077272000	-4.116576000
H	-3.624720000	2.721435000	-2.745984000
H	-4.809094000	1.492835000	-1.535966000
H	-4.807889000	-1.493386000	1.538642000
H	-3.622925000	-2.734345000	2.735455000
H	-2.071954000	-4.090278000	4.104994000
H	0.409245000	-3.784942000	3.803991000
H	1.200478000	-2.113857000	2.123942000
H	-6.693075000	-2.121461000	0.402034000
H	-9.141606000	-2.106230000	0.416473000
H	-9.137243000	2.118415000	-0.385952000
H	-6.688755000	2.125499000	-0.387755000
H	-11.245651000	-0.770410000	0.642542000
H	-11.196199000	-0.207300000	-1.030216000
H	-11.244161000	0.970344000	0.284832000
H	-1.197493000	-2.115299000	-2.125307000
H	-0.403961000	-3.782408000	-3.808268000
H	2.077654000	-4.082391000	-4.111182000
H	3.626760000	-2.725687000	-2.740295000
H	4.810190000	-1.491719000	-1.534728000
H	4.806799000	1.494559000	1.539820000
H	3.620887000	2.729928000	2.741324000
H	2.068905000	4.085001000	4.110554000
H	-0.412074000	3.781447000	3.805946000
H	-1.202068000	2.112730000	2.122987000
H	6.691044000	-2.122364000	-0.387140000
H	9.139489000	-2.112484000	-0.386360000
H	9.139381000	2.112189000	0.415927000
H	6.690793000	2.124620000	0.402539000
H	11.245013000	-0.957516000	0.299745000
H	11.195568000	0.197182000	-1.035138000
H	11.245370000	0.789050000	0.627896000

Bis(4′-(4-methylphenyl)-2,2′:6′,2′′-terpyridine)manganese(III) q = 3, S = 2 (gas phase OLYP-D3)

C	-0.394492000	-2.222949000	-2.235966000
H	-1.448456000	-2.037312000	-2.050573000
C	0.013226000	-3.159683000	-3.182876000
H	-0.726215000	-3.717701000	-3.749323000
C	1.381242000	-3.355881000	-3.379032000
H	1.737930000	-4.077214000	-4.109527000
C	2.290997000	-2.613871000	-2.625627000
H	3.355274000	-2.759372000	-2.771941000
C	1.813404000	-1.689145000	-1.691070000
C	2.664892000	-0.844414000	-0.832055000

C	4.050720000	-0.857880000	-0.841201000
H	4.566113000	-1.512217000	-1.531346000
C	4.804462000	0.000488000	-0.000902000
C	4.050513000	0.857722000	0.840396000
H	4.565866000	1.512749000	1.529939000
C	2.664699000	0.842846000	0.832369000
C	1.812991000	1.686916000	1.691804000
C	2.290321000	2.611319000	2.626815000
H	3.354561000	2.756959000	2.773275000
C	1.380360000	3.352764000	3.380527000
H	1.736843000	4.073839000	4.111375000
C	0.012397000	3.156333000	3.184230000
H	-0.727204000	3.713921000	3.750892000
C	-0.395059000	2.219907000	2.236905000
H	-1.448974000	2.034091000	2.051417000
C	6.258462000	0.001594000	-0.001617000
C	6.991735000	1.145405000	0.404328000
H	6.475558000	2.060386000	0.680428000
C	8.376561000	1.147702000	0.386569000
H	8.912138000	2.049074000	0.672931000
C	9.107290000	0.006453000	-0.009410000
C	8.378193000	-1.136891000	-0.402603000
H	8.915567000	-2.033994000	-0.699061000
C	6.993413000	-1.139834000	-0.411515000
H	6.478842000	-2.054991000	-0.689938000
C	10.602304000	-0.000629000	0.011894000
H	11.015905000	0.999713000	-0.142177000
H	11.016146000	-0.681903000	-0.736772000
H	10.955677000	-0.348566000	0.994148000
N	0.478015000	-1.504492000	-1.507828000
N	1.973825000	-0.001053000	0.000331000
N	0.477650000	1.501990000	1.508467000
Mn	-0.000040000	-0.001101000	0.000142000
C	0.394254000	-2.222851000	2.236327000
H	1.448234000	-2.037273000	2.050965000
C	-0.013555000	-3.159526000	3.183256000
H	0.725839000	-3.717557000	3.749752000
C	-1.381588000	-3.355640000	3.379371000
H	-1.738341000	-4.076921000	4.109885000
C	-2.291275000	-2.613614000	2.625900000
H	-3.355566000	-2.759048000	2.772181000
C	-1.813602000	-1.688956000	1.691317000
C	-2.664997000	-0.844233000	0.832214000
C	-4.050825000	-0.857710000	0.841177000
H	-4.566299000	-1.512028000	1.531277000
C	-4.804471000	0.000609000	0.000737000
C	-4.050417000	0.857816000	-0.840500000
H	-4.565686000	1.512788000	-1.530154000
C	-2.664604000	0.842982000	-0.832273000
C	-1.812790000	1.687066000	-1.691579000
C	-2.289996000	2.611616000	-2.626508000

H	-3.354216000	2.757384000	-2.772978000
C	-1.379940000	3.353065000	-3.380100000
H	-1.736330000	4.074255000	-4.110881000
C	-0.012004000	3.156491000	-3.183770000
H	0.727673000	3.714071000	-3.750340000
C	0.395335000	2.219931000	-2.236526000
H	1.449224000	2.034003000	-2.051017000
C	-6.258463000	0.001689000	0.001251000
C	-6.991713000	1.145445000	-0.404915000
H	-6.475529000	2.060418000	-0.681027000
C	-8.376535000	1.147716000	-0.387356000
H	-8.912083000	2.049052000	-0.673888000
C	-9.107307000	0.006488000	0.008637000
C	-8.378240000	-1.136791000	0.402054000
H	-8.915631000	-2.033874000	0.698536000
C	-6.993457000	-1.139703000	0.411162000
H	-6.478924000	-2.054830000	0.689755000
C	-10.602313000	-0.000572000	-0.012903000
H	-11.015898000	0.999580000	0.142513000
H	-11.016314000	-0.682883000	0.734714000
H	-10.955531000	-0.347011000	-0.995743000
N	-0.478187000	-1.504378000	1.508126000
N	-1.973833000	-0.000878000	-0.000104000
N	-0.477463000	1.502010000	-1.508201000

Bis(4′-(4-methylphenyl)-2,2′:6′,2′′-terpyridine)manganese(II) q = 2, S = 5/2 (solvent phase OLYP-D3)

Mn	-0.000002000	0.000314000	0.002098000
N	-0.704205000	1.544635000	-1.537952000
N	-2.215924000	-0.000677000	0.001156000
N	-0.704083000	-1.545192000	1.540801000
N	0.704138000	-1.544697000	-1.537569000
N	2.215921000	0.000576000	0.001539000
N	0.704147000	1.545316000	1.541002000
C	0.124632000	2.296879000	-2.278208000
C	-0.325874000	3.259568000	-3.179597000
C	-1.701227000	3.448182000	-3.309486000
C	-2.569390000	2.670728000	-2.544763000
C	-2.042053000	1.717034000	-1.664606000
C	-2.889119000	0.838362000	-0.812685000
C	-4.285197000	0.853879000	-0.841771000
C	-5.014916000	-0.000553000	0.001419000
C	-4.285187000	-0.855132000	0.844407000
C	-2.889072000	-0.839691000	0.815043000
C	-2.041888000	-1.718405000	1.666810000
C	-2.569043000	-2.672938000	2.546157000
C	-1.700745000	-3.450372000	3.310755000
C	-0.325445000	-3.260898000	3.181551000
C	0.124890000	-2.297399000	2.280939000
C	-6.491685000	-0.000084000	0.000851000

C	-7.216708000	-1.192973000	0.179859000
C	-8.609647000	-1.188301000	0.174743000
C	-9.334721000	0.002145000	0.002442000
C	-8.608005000	1.191955000	-0.166669000
C	-7.215027000	1.194251000	-0.174889000
C	-10.840112000	-0.001301000	-0.027644000
C	-0.124697000	-2.297038000	-2.277725000
C	0.325791000	-3.259968000	-3.178870000
C	1.701135000	-3.448729000	-3.308623000
C	2.569304000	-2.671176000	-2.544008000
C	2.041978000	-1.717243000	-1.664101000
C	2.889080000	-0.838475000	-0.812314000
C	4.285159000	-0.853931000	-0.841523000
C	5.014915000	0.000585000	0.001550000
C	4.285220000	0.855148000	0.844586000
C	2.889107000	0.839651000	0.815338000
C	2.041961000	1.718358000	1.667148000
C	2.569134000	2.672714000	2.546675000
C	1.700846000	3.450141000	3.311293000
C	0.325535000	3.260844000	3.181933000
C	-0.124824000	2.297519000	2.281151000
C	6.491684000	0.000246000	0.000820000
C	7.215141000	-1.194070000	-0.175039000
C	8.608058000	-1.191662000	-0.166953000
C	9.334721000	-0.001739000	0.002140000
C	8.609595000	1.188586000	0.174513000
C	7.216596000	1.193147000	0.179761000
C	10.840110000	0.001365000	-0.028085000
H	1.188185000	2.118358000	-2.141090000
H	0.386055000	3.841469000	-3.756402000
H	-2.097871000	4.191027000	-3.996005000
H	-3.638952000	2.815591000	-2.636941000
H	-4.814314000	1.502712000	-1.526793000
H	-4.814119000	-1.503845000	1.529673000
H	-3.638562000	-2.818476000	2.637772000
H	-2.097242000	-4.193879000	3.996642000
H	0.386573000	-3.842743000	3.758302000
H	1.188393000	-2.118166000	2.144365000
H	-6.689916000	-2.136374000	0.294951000
H	-9.144102000	-2.127089000	0.303503000
H	-9.141011000	2.132767000	-0.285895000
H	-6.686858000	2.137692000	-0.283190000
H	-11.250551000	-0.807423000	0.588131000
H	-11.203400000	-0.155378000	-1.052725000
H	-11.249120000	0.950877000	0.323651000
H	-1.188248000	-2.118412000	-2.140734000
H	-0.386149000	-3.841932000	-3.755597000
H	2.097765000	-4.191762000	-3.994947000
H	3.638861000	-2.816149000	-2.636068000
H	4.814237000	-1.502775000	-1.526564000
H	4.814186000	1.503903000	1.529784000

H	3.638659000	2.818120000	2.638422000
H	2.097361000	4.193509000	3.997320000
H	-0.386470000	3.842698000	3.758691000
H	-1.188332000	2.118424000	2.144428000
H	6.687021000	-2.137542000	-0.283314000
H	9.141144000	-2.132431000	-0.286248000
H	9.143984000	2.127406000	0.303242000
H	6.689759000	2.136515000	0.294917000
H	11.249055000	-0.945415000	0.337761000
H	11.203348000	0.139160000	-1.055497000
H	11.250655000	0.816734000	0.575211000

Bis(4′-(4-methylphenyl)-2,2′:6′,2′′-terpyridine)manganese(III) q = 3, S = 2 (solvent phase OLYP-D3)

C	-0.393235000	-2.230129000	-2.219353000
H	-1.446038000	-2.034598000	-2.044627000
C	0.017173000	-3.185926000	-3.146151000
H	-0.724521000	-3.745943000	-3.705904000
C	1.383116000	-3.397726000	-3.325887000
H	1.740150000	-4.137053000	-4.036683000
C	2.295242000	-2.650149000	-2.581696000
H	3.358727000	-2.810342000	-2.710717000
C	1.815251000	-1.703924000	-1.673184000
C	2.665708000	-0.848737000	-0.827676000
C	4.054238000	-0.855367000	-0.843874000
H	4.573362000	-1.506244000	-1.533459000
C	4.789181000	0.000905000	-0.000884000
C	4.052745000	0.854144000	0.843920000
H	4.571113000	1.506383000	1.532777000
C	2.664304000	0.843857000	0.829386000
C	1.812610000	1.697433000	1.675279000
C	2.291273000	2.643216000	2.584980000
H	3.354524000	2.804000000	2.715111000
C	1.378125000	3.389548000	3.329144000
H	1.734171000	4.128458000	4.040868000
C	0.012464000	3.177052000	3.148160000
H	-0.730032000	3.736143000	3.707779000
C	-0.396595000	2.221719000	2.220304000
H	-1.449171000	2.025749000	2.044698000
C	6.256882000	0.004611000	-0.002069000
C	6.978843000	1.177478000	0.299376000
H	6.453355000	2.105616000	0.503690000
C	8.369656000	1.179200000	0.286500000
H	8.901028000	2.103284000	0.500835000
C	9.098197000	0.013939000	-0.006997000
C	8.375714000	-1.156138000	-0.301838000
H	8.912562000	-2.075002000	-0.525831000
C	6.985836000	-1.163689000	-0.308480000
H	6.465294000	-2.093607000	-0.517274000
C	10.601396000	0.007959000	0.017647000

H	11.009087000	1.014681000	-0.108700000
H	11.009781000	-0.639998000	-0.764340000
H	10.963692000	-0.378442000	0.979908000
N	0.481470000	-1.508800000	-1.501038000
N	1.985727000	-0.003142000	0.000986000
N	0.479088000	1.501478000	1.502063000
Mn	0.000002000	-0.003638000	-0.000001000
C	0.393233000	-2.230154000	2.219330000
H	1.446037000	-2.034623000	2.044607000
C	-0.017176000	-3.185962000	3.146117000
H	0.724518000	-3.745987000	3.705864000
C	-1.383119000	-3.397763000	3.325850000
H	-1.740155000	-4.137098000	4.036638000
C	-2.295244000	-2.650175000	2.581667000
H	-3.358730000	-2.810370000	2.710687000
C	-1.815251000	-1.703940000	1.673167000
C	-2.665705000	-0.848742000	0.827668000
C	-4.054236000	-0.855371000	0.843870000
H	-4.573360000	-1.506251000	1.533452000
C	-4.789179000	0.000905000	0.000885000
C	-4.052743000	0.854148000	-0.843915000
H	-4.571111000	1.506391000	-1.532769000
C	-2.664301000	0.843862000	-0.829380000
C	-1.812610000	1.697448000	-1.675264000
C	-2.291276000	2.643240000	-2.584955000
H	-3.354527000	2.804025000	-2.715084000
C	-1.378129000	3.389583000	-3.329111000
H	-1.734177000	4.128500000	-4.040827000
C	-0.012467000	3.177087000	-3.148129000
H	0.730028000	3.736185000	-3.707743000
C	0.396592000	2.221744000	-2.220283000
H	1.449169000	2.025774000	-2.044679000
C	-6.256882000	0.004612000	0.002070000
C	-6.978842000	1.177480000	-0.299370000
H	-6.453354000	2.105618000	-0.503681000
C	-8.369656000	1.179201000	-0.286495000
H	-8.901028000	2.103285000	-0.500826000
C	-9.098196000	0.013939000	0.006999000
C	-8.375713000	-1.156140000	0.301835000
H	-8.912560000	-2.075004000	0.525824000
C	-6.985834000	-1.163690000	0.308477000
H	-6.465293000	-2.093609000	0.517267000
C	-10.601395000	0.007959000	-0.017641000
H	-11.009086000	1.014684000	0.108682000
H	-11.009778000	-0.639977000	0.764365000
H	-10.963696000	-0.378468000	-0.979890000
N	-0.481471000	-1.508817000	1.501025000
N	-1.985725000	-0.003143000	-0.000987000
N	-0.479090000	1.501495000	-1.502051000

4. PW6B95D3 Optimized Cartesian coordinates (Å)

Bis(terpyridine)manganese(II) q = 2, S = 5/2 (gas phase PW6B95D3)

Mn	0.000000000	0.000000000	0.000000000
N	-2.147029000	0.000000000	0.694570000
C	-2.368779000	0.000000000	2.014961000
C	-3.654620000	0.000000000	2.527887000
C	-4.728103000	0.000000000	1.657539000
C	-4.491859000	0.000000000	0.296801000
C	-3.181513000	0.000000000	-0.137621000
N	0.000000000	0.000000000	2.215907000
C	-1.161188000	0.000000000	2.869258000
C	-1.195794000	0.000000000	4.254629000
C	0.000000000	0.000000000	4.945846000
C	1.195794000	0.000000000	4.254629000
C	1.161188000	0.000000000	2.869258000
N	2.147029000	0.000000000	0.694570000
C	2.368779000	0.000000000	2.014961000
C	3.654620000	0.000000000	2.527887000
C	4.728103000	0.000000000	1.657539000
C	4.491859000	0.000000000	0.296801000
C	3.181513000	0.000000000	-0.137621000
N	0.000000000	2.147029000	-0.694570000
C	0.000000000	2.368779000	-2.014961000
C	0.000000000	3.654620000	-2.527887000
C	0.000000000	4.728103000	-1.657539000
C	0.000000000	4.491859000	-0.296801000
C	0.000000000	3.181513000	0.137621000
N	0.000000000	0.000000000	-2.215907000
C	0.000000000	1.161188000	-2.869258000
C	0.000000000	1.195794000	-4.254629000
C	0.000000000	0.000000000	-4.945846000
C	0.000000000	-1.195794000	-4.254629000
C	0.000000000	-1.161188000	-2.869258000
N	0.000000000	-2.147029000	-0.694570000
C	0.000000000	-2.368779000	-2.014961000
C	0.000000000	-3.654620000	-2.527887000
C	0.000000000	-4.728103000	-1.657539000
C	0.000000000	-4.491859000	-0.296801000
C	0.000000000	-3.181513000	0.137621000
H	-3.826457000	0.000000000	3.589381000
H	-5.735195000	0.000000000	2.040244000
H	-5.299494000	0.000000000	-0.414541000
H	-2.947459000	0.000000000	-1.191323000
H	-2.127552000	0.000000000	4.790658000
H	0.000000000	0.000000000	6.023226000
H	2.127552000	0.000000000	4.790658000
H	3.826457000	0.000000000	3.589381000
H	5.735195000	0.000000000	2.040244000

H	5.299494000	0.000000000	-0.414541000
H	2.947459000	0.000000000	-1.191323000
H	0.000000000	3.826457000	-3.589381000
H	0.000000000	5.735195000	-2.040244000
H	0.000000000	5.299494000	0.414541000
H	0.000000000	2.947459000	1.191323000
H	0.000000000	2.127552000	-4.790658000
H	0.000000000	0.000000000	-6.023226000
H	0.000000000	-2.127552000	-4.790658000
H	0.000000000	-3.826457000	-3.589381000
H	0.000000000	-5.735195000	-2.040244000
H	0.000000000	-5.299494000	0.414541000
H	0.000000000	-2.947459000	1.191323000

Bis(terpyridine)manganese(III) q = 3, S = 2 (gas phase PW6B95D3)

Mn	0.000000000	0.000000000	0.079498000
N	0.000000000	0.000000000	-1.958175000
N	0.000000000	2.181145000	-0.471900000
N	2.040494000	0.000000000	0.499861000
N	0.000000000	-2.181145000	-0.471900000
N	-2.040494000	0.000000000	0.499861000
N	0.000000000	0.000000000	2.049480000
C	0.000000000	0.000000000	-4.698756000
C	0.000000000	1.194530000	-4.005364000
C	0.000000000	3.672250000	-2.320424000
C	0.000000000	-1.194530000	-4.005364000
C	0.000000000	4.752663000	-1.455482000
C	0.000000000	1.174979000	-2.622955000
C	0.000000000	2.392412000	-1.795956000
C	0.000000000	-1.174979000	-2.622955000
C	4.352096000	0.000000000	-0.036251000
C	0.000000000	4.526615000	-0.092554000
C	3.020519000	0.000000000	-0.401030000
C	0.000000000	-3.672250000	-2.320424000
C	0.000000000	-2.392412000	-1.795956000
C	4.673188000	0.000000000	1.307412000
C	0.000000000	3.220427000	0.356302000
C	0.000000000	-4.752663000	-1.455482000
C	-3.020519000	0.000000000	-0.401030000
C	3.655072000	0.000000000	2.247024000
C	2.345033000	0.000000000	1.816689000
C	-4.352096000	0.000000000	-0.036251000
C	0.000000000	-4.526615000	-0.092554000
C	0.000000000	-3.220427000	0.356302000
C	1.178157000	0.000000000	2.700706000
C	-2.345033000	0.000000000	1.816689000
C	-4.673188000	0.000000000	1.307412000
C	-1.178157000	0.000000000	2.700706000
C	-3.655072000	0.000000000	2.247024000
C	1.201229000	0.000000000	4.081671000

C	-1.201229000	0.000000000	4.081671000
C	0.000000000	0.000000000	4.770109000
H	0.000000000	0.000000000	-5.776649000
H	0.000000000	2.127315000	-4.539581000
H	0.000000000	3.840303000	-3.382662000
H	0.000000000	5.757293000	-1.845406000
H	0.000000000	-2.127315000	-4.539581000
H	5.115125000	0.000000000	-0.795795000
H	2.728300000	0.000000000	-1.437793000
H	0.000000000	-3.840303000	-3.382662000
H	0.000000000	5.341040000	0.611566000
H	5.703000000	0.000000000	1.625394000
H	-2.728300000	0.000000000	-1.437793000
H	0.000000000	-5.757293000	-1.845406000
H	0.000000000	2.999175000	1.412214000
H	-5.115125000	0.000000000	-0.795795000
H	3.887390000	0.000000000	3.297999000
H	0.000000000	-5.341040000	0.611566000
H	0.000000000	-2.999175000	1.412214000
H	-5.703000000	0.000000000	1.625394000
H	2.134840000	0.000000000	4.616502000
H	-3.887390000	0.000000000	3.297999000
H	-2.134840000	0.000000000	4.616502000
H	0.000000000	0.000000000	5.848142000

Bis(4′-(4-methylphenyl)-2,2′:6′,2′′-terpyridine)manganese(II) q = 2, S = 5/2 (gas phase PW6B95D3)

Mn	3.062999000	2.173824000	21.065132000
N	4.083739000	0.292111000	20.342762000
N	3.310290000	2.333599000	18.886637000
N	2.205260000	4.160420000	20.411918000
N	1.067473000	1.185131000	21.453040000
N	2.800631000	2.011914000	23.241621000
N	4.893160000	3.057669000	22.052630000
C	4.440795000	-0.699429000	21.150273000
C	5.054840000	-1.848823000	20.696700000
C	5.304534000	-1.963071000	19.342743000
C	4.935433000	-0.933316000	18.499151000
C	4.322813000	0.189703000	19.029788000
C	3.892352000	1.343392000	18.208089000
C	4.060521000	1.411397000	16.840585000
C	3.621334000	2.533511000	16.137465000
C	3.018557000	3.550535000	16.878153000
C	2.877698000	3.419827000	18.244315000
C	2.258453000	4.451665000	19.106545000
C	1.759451000	5.648058000	18.619093000
C	1.198602000	6.553868000	19.498428000
C	1.147537000	6.245264000	20.844051000
C	1.664740000	5.033364000	21.253648000
C	3.785994000	2.638147000	14.690739000

C	2.879767000	3.361547000	13.917708000
C	3.036404000	3.453508000	12.552266000
C	4.106544000	2.841637000	11.907388000
C	5.015805000	2.130091000	12.683301000
C	4.856804000	2.020622000	14.047248000
C	4.261357000	2.927259000	10.425473000
C	0.248127000	0.791952000	20.485205000
C	-0.966178000	0.185706000	20.733883000
C	-1.336128000	-0.016089000	22.049549000
C	-0.486826000	0.392100000	23.059658000
C	0.716383000	0.993721000	22.730374000
C	1.690962000	1.464501000	23.740349000
C	1.495394000	1.349897000	25.101098000
C	2.467070000	1.813650000	25.988289000
C	3.614227000	2.381081000	25.432990000
C	3.750111000	2.463762000	24.062595000
C	4.928322000	3.057008000	23.390691000
C	6.008939000	3.583264000	24.078656000
C	7.066384000	4.117392000	23.368127000
C	7.022161000	4.113670000	21.987317000
C	5.911822000	3.571225000	21.373450000
C	2.290149000	1.709766000	27.433612000
C	1.018569000	1.752607000	28.002243000
C	0.855153000	1.665226000	29.367194000
C	1.947888000	1.513706000	30.214784000
C	3.215521000	1.467214000	29.643721000
C	3.387686000	1.569893000	28.280816000
C	1.764009000	1.382277000	31.690008000
H	4.223682000	-0.562352000	22.198759000
H	5.326915000	-2.628309000	21.387048000
H	5.781333000	-2.843598000	18.945142000
H	5.124421000	-1.011345000	17.443518000
H	4.504618000	0.594318000	16.301817000
H	2.692779000	4.443467000	16.376519000
H	1.805015000	5.877759000	17.569650000
H	0.806867000	7.489319000	19.134793000
H	0.719082000	6.923596000	21.561334000
H	1.648885000	4.745647000	22.293961000
H	2.023663000	3.826531000	14.380422000
H	2.315367000	4.006329000	11.971279000
H	5.864633000	1.664003000	12.208733000
H	5.597462000	1.486075000	14.620774000
H	3.848828000	3.852021000	10.036624000
H	3.733475000	2.106877000	9.942630000
H	5.303261000	2.860882000	10.131482000
H	0.582800000	0.971585000	19.474762000
H	-1.599271000	-0.117742000	19.918217000
H	-2.275341000	-0.486283000	22.289454000
H	-0.764046000	0.239733000	24.087329000
H	0.610116000	0.880739000	25.490044000
H	4.373131000	2.775767000	26.083669000

H	6.032193000	3.580987000	25.153656000
H	7.913911000	4.531297000	23.888830000
H	7.825220000	4.520010000	21.397243000
H	5.829394000	3.544830000	20.297441000
H	0.151336000	1.899363000	27.378054000
H	-0.136000000	1.724344000	29.788032000
H	4.077621000	1.344970000	30.280006000
H	4.383031000	1.503313000	27.870816000
H	0.883800000	1.918129000	32.028608000
H	1.633461000	0.336585000	31.962618000
H	2.626938000	1.753994000	32.231864000

Bis(4′-(4-methylphenyl)-2,2′:6′,2′′-terpyridine)manganese(III) q = 3, S = 2 (gas phase PW6B95D3)

C	-3.407875000	1.616369000	13.505197000
H	-4.349118000	1.813120000	13.018095000
C	-2.522230000	0.688128000	12.998661000
H	-2.765182000	0.144765000	12.101858000
C	-1.329928000	0.479973000	13.666829000
H	-0.614082000	-0.236386000	13.298795000
C	-1.062136000	1.201507000	14.815951000
H	-0.138186000	1.050430000	15.345777000
C	-1.994716000	2.115017000	15.267691000
C	-1.838613000	2.942473000	16.469095000
C	-0.755567000	2.916792000	17.306551000
H	0.068576000	2.265121000	17.082073000
C	-0.689698000	3.759663000	18.430695000
C	-1.790507000	4.611892000	18.631750000
H	-1.812567000	5.257213000	19.490475000
C	-2.855643000	4.602152000	17.771321000
C	-4.053883000	5.438240000	17.904235000
C	-4.265964000	6.350619000	18.919574000
H	-3.531423000	6.495038000	19.692099000
C	-5.440930000	7.079959000	18.933270000
H	-5.624399000	7.795593000	19.717660000
C	-6.373553000	6.880441000	17.932400000
H	-7.298918000	7.429739000	17.910012000
C	-6.096763000	5.953420000	16.949101000
H	-6.793700000	5.763686000	16.148955000
C	0.440330000	3.749628000	19.325751000
C	0.717400000	4.846945000	20.153222000
H	0.110462000	5.736160000	20.104232000
C	1.802008000	4.838421000	20.993899000
H	2.011839000	5.704147000	21.600933000
C	2.648349000	3.730769000	21.069465000
C	2.369128000	2.634194000	20.251754000
H	3.005412000	1.765367000	20.300443000
C	1.298768000	2.643223000	19.393206000
H	1.104668000	1.762851000	18.802774000
C	3.799796000	3.710119000	22.009466000

H	4.182153000	4.707790000	22.194273000
H	4.604152000	3.082671000	21.641796000
H	3.484039000	3.301615000	22.969271000
N	-3.151470000	2.312123000	14.609679000
N	-2.878446000	3.776094000	16.700449000
N	-4.967523000	5.250261000	16.934932000
Mn	-4.410362000	3.782071000	15.496609000
C	-5.486031000	1.583400000	17.485164000
H	-4.550657000	1.765477000	17.989947000
C	-6.389168000	0.661114000	17.971794000
H	-6.167315000	0.108612000	18.868495000
C	-7.572535000	0.471315000	17.282528000
H	-8.302544000	-0.239376000	17.633287000
C	-7.812705000	1.204157000	16.134971000
H	-8.730040000	1.066256000	15.590663000
C	-6.861266000	2.110581000	15.705695000
C	-6.995060000	2.946320000	14.504816000
C	-8.073872000	2.919637000	13.661048000
H	-8.898576000	2.267681000	13.881437000
C	-8.136098000	3.759725000	12.536263000
C	-7.035480000	4.611447000	12.341346000
H	-7.010162000	5.255683000	11.482159000
C	-5.973671000	4.604095000	13.206828000
C	-4.781070000	5.449901000	13.062726000
C	-4.588572000	6.358435000	12.038695000
H	-5.330380000	6.491548000	11.271334000
C	-3.422234000	7.100348000	12.009509000
H	-3.253935000	7.812881000	11.218941000
C	-2.478568000	6.917578000	13.003158000
H	-1.559220000	7.477256000	13.013713000
C	-2.738186000	5.993251000	13.993878000
H	-2.031291000	5.816718000	14.788879000
C	-9.261902000	3.747120000	11.634922000
C	-9.537903000	4.844199000	10.807352000
H	-8.933623000	5.735022000	10.860410000
C	-10.618217000	4.833053000	9.960837000
H	-10.827452000	5.698675000	9.353427000
C	-11.460670000	3.723055000	9.879881000
C	-11.182346000	2.626787000	10.697938000
H	-11.815740000	1.756069000	10.645363000
C	-10.116230000	2.638330000	11.562076000
H	-9.922408000	1.758211000	12.153039000
C	-12.607364000	3.699761000	8.933747000
H	-12.992008000	4.696449000	8.748372000
H	-13.411694000	3.069624000	9.296859000
H	-12.285759000	3.293356000	7.975071000
N	-5.715359000	2.290026000	16.382179000
N	-5.952412000	3.779797000	14.280306000
N	-3.858812000	5.277727000	14.023238000
